# The MYO1B and MYO5B motor proteins and the sorting nexin SNX27 regulate apical targeting of membrane mucin MUC17 in enterocytes

**DOI:** 10.1042/BCJ20240204

**Published:** 2025-01-08

**Authors:** Sofia Jäverfelt, Gustaf Hellsén, Izumi Kaji, James R. Goldenring, Thaher Pelaseyed

**Affiliations:** 1 Department of Medical Biochemistry and Cell Biology, Institute of Biomedicine, University of Gothenburg, Box 440, 405 30 Gothenburg, Sweden;; 2 Epithelial Biology Center, Vanderbilt University Medical Center; Section of Surgical Sciences, Vanderbilt University Medical Center, Nashville, TN 37232, U.S.A;; 3 Nashville VA Medical Center, Nashville, TN 37232, U.S.A;; 4 Department of Cell and Developmental Biology, Vanderbilt University, Nashville, TN 37232, U.S.A

## Abstract

A dense glycocalyx, composed of the megaDalton-sized membrane mucin MUC17, coats the microvilli in the apical brush border of transporting intestinal epithelial cells, called enterocytes. The formation of the MUC17-based glycocalyx in the mouse small intestine occurs at the critical suckling-weaning transition. The glycocalyx extends 1 μm into the intestinal lumen and prevents the gut bacteria from directly attaching to the enterocytes. To date, the mechanism behind the positioning of MUC17 to the brush border is not known. Here, we show that the actin-based motor proteins MYO1B and MYO5B, and the sorting nexin SNX27, regulate apical targeting of MUC17 in enterocytes. We demonstrate that MUC17 turnover at the brush border is slow and controlled by MYO1B and SNX27. Furthermore, we report that MYO1B regulates MUC17 protein levels in enterocytes, whereas MYO5B specifically governs MUC17 levels at the brush border. Together, our results extend our understanding of the apical targeting of membrane mucins and provide mechanistic insights into how defective positioning of MUC17 renders enterocytes sensitive to bacterial challenges.

## Introduction

The epithelium of the small intestine consists of a tight single layer of highly polarized epithelial cells covered by a loose and permeable mucus layer [1,2]. Luminal bacteria that breach the mucus layer encounter a second line of defense in a 1 μm thick glycocalyx attached to the microvillus-studded apical brush border of transporting intestinal epithelial cells, called enterocytes [3]. Our previous studies identified membrane mucin MUC17 as a major component of the enterocytic glycocalyx [4], which forms a physical barrier that prevents direct bacterial contact with enterocytes [5]. MUC17 is a 4493 amino acids long protein with an extracellular proline, threonine, serine (PTS)-rich domain consisting of recurring proline, threonine, and serine residues organized in 60 tandem repeats (TRs) [6]. The serine and threonine residues undergo *O*-linked glycosylation, resulting in a glycosylated mucin domain comprising 80% of the mucin’s molecular weight [7]. The mucin domain connects to a transmembrane domain via an evolutionarily conserved sea urchin sperm protein, enterokinase, and agrin domain that is auto-catalytically cleaved during mucin biosynthesis and serves as a mechanosensor at the cell surface [8–10]. The cytoplasmic tail domain of MUC17 harbors a class I PSD95, DLG1, ZO-1 (PDZ)-binding motif (PBM) [6], which allows PDZ-containing protein 1 (PDZK1) to retain MUC17 in the apical membrane. In addition, MUC17 holds two phosphorylation sites with yet undefined functions [11].

The MUC17-based glycocalyx is replenished every 12–24 hours [12], which is considerably faster than the turnover of individual enterocytes (three to five days) [13–15]. As a result, enterocytes must carefully regulate the turnover of MUC17 to guarantee the barrier integrity of the glycocalyx. MUC17 turnover is slower in the ileum of germ-free mice compared with colonized mice, suggesting that the commensal gut microbiota plays a role in the renewal of the glycocalyx [16]. Recycling of membrane proteins in enterocytes is a tightly regulated process that dictates the protein composition, surface receptor activity, and ion transport at the apical cell surface. Membrane protein trafficking to and from the cell surface is mediated by myosin motor proteins and Rab GTPases, which coordinate the transport and retention of vesicle-borne membrane proteins within specific endosomal compartments [17]. In addition, sorting nexins in early endosomes mediate rapid recycling of proteins back to the plasma membrane [18]. However, how these processes regulate the apical targeting of MUC17 in enterocytes is undefined.

Here, we combined quantitative proteomics, protein-protein interaction assays, CRISPR-Cas9-mediated gene deletion, and imaging to demonstrate that the myosin motor proteins MYO1B and MYO5B regulate MUC17 protein levels and targeting the apical brush border. Moreover, we identified the sorting nexin SNX27 as a novel MUC17 interaction partner.

## Results

### A recombinant MUC17 localizes to the plasma membrane independent of epithelial cell differentiation and brush border maturation

**Figure 1:**
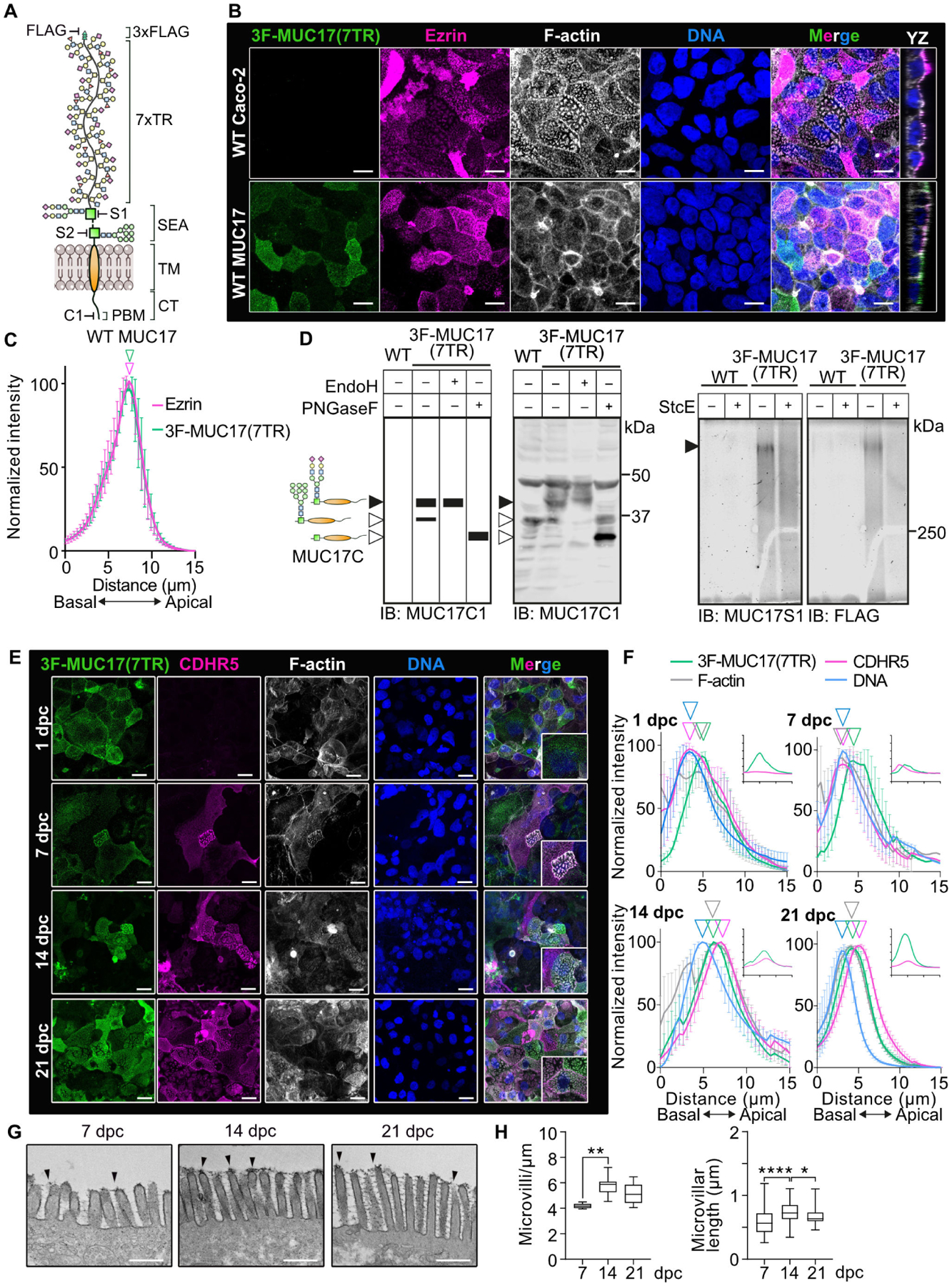
3F-MUC17(7TR) localizes apically in Caco-2 cells. (**A**) Schematic representation of recombinant 3xF-MUC17(7TR). FLAG, MUC17-S1, MUC17-S2, and MUC17-C1 indicate epitope positions for the fragment-specific antibodies. (**B**) Confocal images of Caco-2 cells differentiated for 14 days post confluency (dpc) stained for 3F-MUC17(7TR), Ezrin, F-actin, and nuclear DNA. XZ view represents the orthogonal view of the XY maximum projections. Scale bars 10 μm. (**C**) Normalized intensity profiles of 3F-MUC17(7TR) distribution in relation to Ezrin in WT Caco-2 cells. The vertical arrowheads point to maximum intensity of each protein along the length of the cell; *n* = 3 scans and a mean of 73 cells per group. Data are presented as mean ± SD. (**D**) Enzymatic treatment to assess the *N*- and *O*-glycosylation of 3F-MUC17(7TR). Expected and obtained bands from lysates of WT and 3F-MUC17(7TR)-expressing Caco-2 cells treated with EndoH and PNGaseF analyzed by immunoblotting and probed with MUC17-C1 antibody (left). Black bands represent specific bands. Lysates of WT and 3F-MUC17(7TR)-expressing Caco-2 cells treated with StcE analyzed by in-gel western and probed with FLAG and MUC17-S1 antibodies (right). Filled arrowheads point to fully mature protein. Empty arrowheads point to the immature protein. (**E**) Assessment of 3F-MUC17(7TR) localization during cell differentiation at 1, 7, 14, and 21 dpc. Confocal images represent maximum projections of the entire length of the cell, scale bars 10 μm; *n* = 3 scans with a mean of 60 cells per time point. Insets represent higher magnification of the merged image. (**F**) Normalized intensity profiles of relative 3F-MUC17(7TR) distribution in relation to Cadherin-related family member 5 (CDHR5) and F-actin at 1, 7, 14, and 21 dpc. The vertical arrowheads point to maximum intensity of each protein along the length of the cell; *n* = 3 scans with a mean of 60 cells per time point. Data are presented as mean ± SD. Absolute intensities of MUC17 (green) and CDHR5 (magenta) are shown in insets. (**G**) Transmission electron microscopy (TEM) micrographs of the glycocalyx at 7, 14, and 21 dpc. The arrowheads point to the glycocalyx in the brush border. Scale bars 500 nm. (**H**) Measurements of microvillar density and microvillar length in TEM micrographs; *n* = 6 cells for each group. Data are presented as mean ± SD. *P < 0.05, **P < 0.005, and ****P < 0.0001 as determined by one-way ANOVA followed by Tukey’s multiple comparisons test. CT, cytoplasmic tail domain; PBM, PDZ-binding motif; SEA, sea urchin sperm protein, enterokinase, and agrin; TM, transmembrane domain; TR, tandem repeat.

To identify proteins responsible for the apical targeting of MUC17, we turned to the Caco-2 colorectal adenocarcinoma cell line. Unpolarized Caco-2 cells differentiate over 21 days to form a polarized monolayer of columnar epithelial cells with a defined brush border membrane [19]. Importantly, Caco-2 cells express negligible levels of endogenous MUC17 [11,20], allowing us to introduce constitutive expression of a recombinant MUC17 with an endogenous signal sequence, an N-terminal 3xFlag tag, and a mucin domain consisting of seven PTS-rich TRs (Figure 1A). Immunofluorescence imaging and quantification of protein distribution in the brush border confirmed that 3F-MUC17(7TR) localized to the tip of Ezrin-positive microvilli in differentiated Caco-2 cells (Figure 1B–C), thereby recapitulating the position of the endogenous MUC17 in human and murine enterocytes [5]. Next, we asked whether 3F-MUC17(7TR) was correctly processed by investigating the presence of mature *N*- and *O*-glycans (Figure 1D). Boiling before SDS-PAGE dissociates 3F-MUC17(7TR) into a larger N- and a smaller C-terminal subunit, each detectable with fragment-specific antibodies (Figure 1A). SDS-PAGE separated the C-terminal subunit into two distinct bands; an upper band representing the mature C-terminus carrying EndoH-resistant, PNGaseF-sensitive *N*-glycans and a lower band representing the ER-resident, EndoH-sensitive C-terminus (Figure 1D, left panel). For *O*-glycan analysis, we took advantage of StcE, a bacterial metalloprotease with high specificity for *O*-glycosylated mucin domains [21]. We observed a broad 450 kDa band that was efficiently digested by StcE, thereby validating that the 3F-MUC17(7TR) N-terminus is extensively *O*-glycosylated in Caco-2 cells (Figure 1D, right panel).

Brush border morphology and protein composition change during Caco-2 cell differentiation [22,23], starting from sparsely spread individual microvilli that ultimately coalesce into tightly packed microvilli, marked by the emergence of an intermicrovillar adhesion complex (IMAC) including Cadherin-related family member 2 (CDHR2) and CDHR5 at the tip of microvilli [24]. Since 3F-MUC17(7TR) localized to microvillus tips, we asked whether 3F-MUC17(7TR) localization was influenced by cell differentiation and microvillar packing. Assessment of the localization of 3F-MUC17(7TR) and CDHR5 during cell differentiation revealed robust cell surface staining of 3F-MUC17(7TR) independent of the cell differentiation stage, whereas CDHR5 appeared after seven days of differentiation (Figure 1E–F). Transmission electron microscopy on Caco-2 cells expressing 3F-MUC17(7TR) showed that microvillus length and packing, as well as density of the glycocalyx at microvillar tips, increased at later stages of cell differentiation (Figure 1G–H). Hence, we concluded that 3F-MUC17(7TR) localizes to the cell surface independent of the differentiation state of epithelial cells.

### The interactome of recombinant MUC17 includes mediators of intracellular trafficking

**Figure 2:**
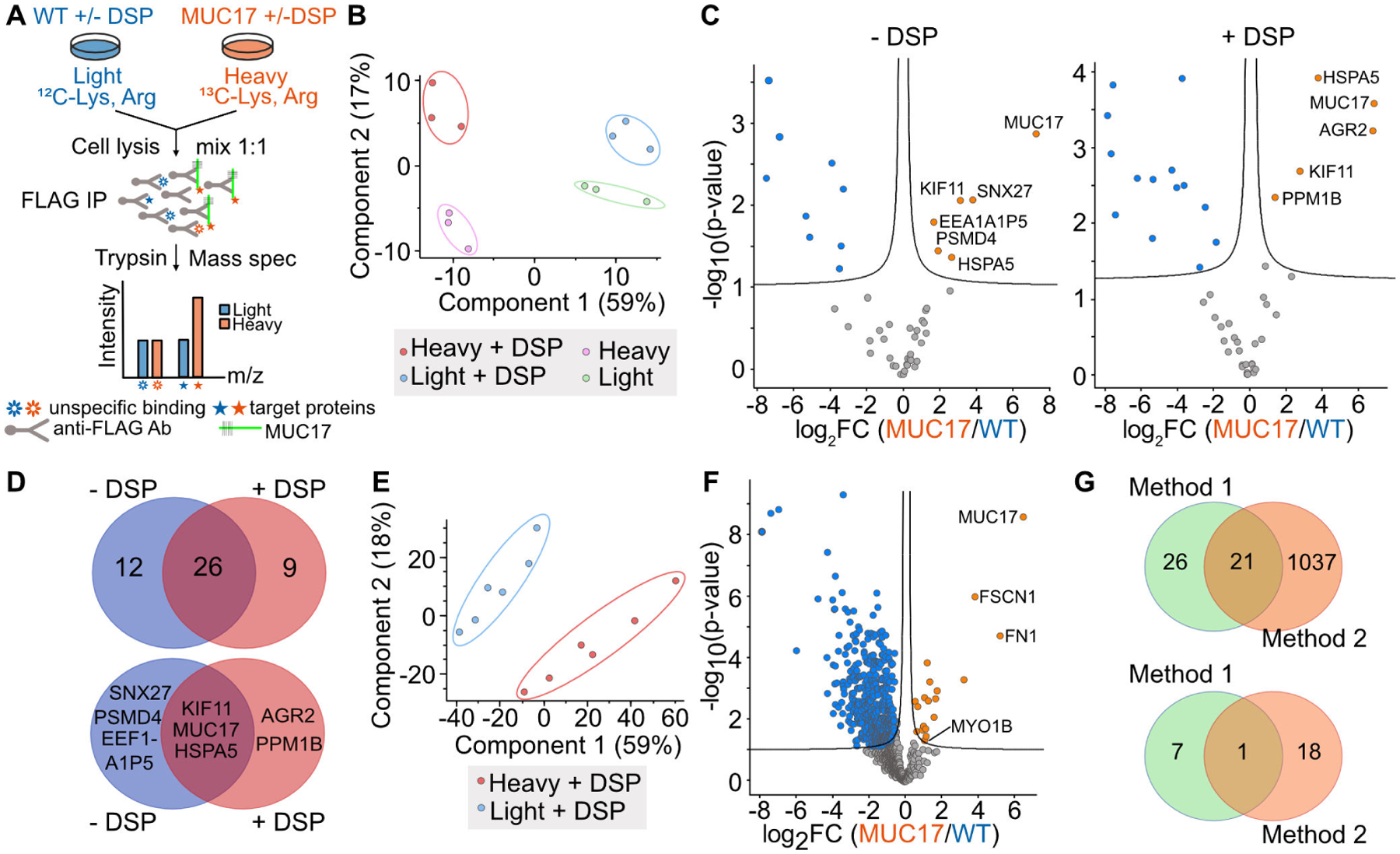
Exploring the interactome of 3F-MUC17(7TR) using quantitative proteomics. (**A**) Schematic representation of the workflow for identifying 3F-MUC17(7TR) interaction partners. (**B**) Principal component analysis (PCA) plot of 3F-MUC17(7TR) (heavy) and WT (light) samples treated with or without the crosslinker DSP and lysed in IGEPAL (method 1). (**C**) Volcano plot of 3F-MUC17(7TR) (red) and WT (blue) samples prepared by method 1; *n* = 3 for each group. Proteins with significantly different abundance (fold change ≥ 2, padj < 0.01) are highlighted in blue for WT and red for 3F-MUC17(7TR)-expressing cells. Specific proteins are labeled. (**D**) Comparisons of all identified proteins (upper) and significantly enriched proteins (lower) identified in (**C**). (**E**) PCA plot of crosslinked 3F-MUC17(7TR) (heavy) and WT (light) samples treated with or without the cross-linker DSP and lysed in SDS (method 2). (**F**) Volcano plot of 3F-MUC17(7TR) (red) and WT (blue) samples prepared by method 2; *n* = 6 for each group. Proteins with significantly different abundance (fold change ≥ 2, padj < 0.01) are highlighted in blue for WT and red for 3F-MUC17(7TR)-expressing cells. Specific proteins are labeled. (G) Comparison of methods 1 and 2 based on all proteins identified (upper) and significantly enriched proteins (lower) identified in (**F**).

To identify proteins that participate in the apical targeting of MUC17, we employed unbiased quantitative proteomics using stable isotope labeling in cell culture (SILAC) and reversible cross-link immunoprecipitation (Re-CLIP) in lysis buffer containing the non-ionic detergent IGEPAL [25] (Figure 2A, Table S1). The addition of the reversible cross-linker dithiobis(succinimidyl propionate) (DSP) enabled us to capture weak and transient protein interactions. Caco-2 (light) and Caco-2–3F-MUC17(7TR) (heavy, C^13^ Lys, C^13^ Arg) cells at 21 days post-confluence (dpc), with or without DSP cross-linking, were subjected to FLAG immunoprecipitation and proteomic identification of the co-precipitated proteins. Thirty-five and 38 confident proteins (fold change ≥ 2, padj < 0.01) were identified in the non-cross-linked and cross-linked samples, respectively (Figure 2B–C, Table S1). Out of these proteins, a minority were significantly enriched with 3F-MUC17(7TR) and included proteins involved in protein recycling (SNX27), processing (PPM1B, HSPA5, PSMD4, EEF1A1P5, AGR2) and transport (KIF11). PPM1B and AGR2 were only identified in cross-linked samples, whereas SNX27, PSMD4, and EEF1A1P5 were only present in non-crosslinked samples. HSPA5 and KIF11 were found in both conditions (Figure 2D). None of the identified proteins have been previously reported as MUC17 interaction partners. Due to the small number of identified proteins, we developed a second Re-CLIP protocol based on the ionic detergent SDS to gain a deeper insight into the interactome of 3F-MUC17(7TR). Using this method, we identified 1058 confident proteins (fold change ≥ 2, padj<0.01), of which 19 proteins were specifically enriched for 3F-MUC17(7TR) (Figure 2E and F, Table S1). Among these, we identified the myosin motor protein MYO1B, the F-actin bundling protein FSCN1, and proteins associated with the secretory pathway (CKAP4, DPYSL2, HM13, RTN4). Apart from the bait 3F-MUC17(7TR), we observed differences between the significantly enriched proteins captured by the two Re-CLIP protocols, suggesting that the two methods identify distinct interaction profiles for 3F-MUC17(7TR) (Figure 2G).

### MYO1B and SNX27 localize with recombinant MUC17 to the apical brush border

**Figure 3:**
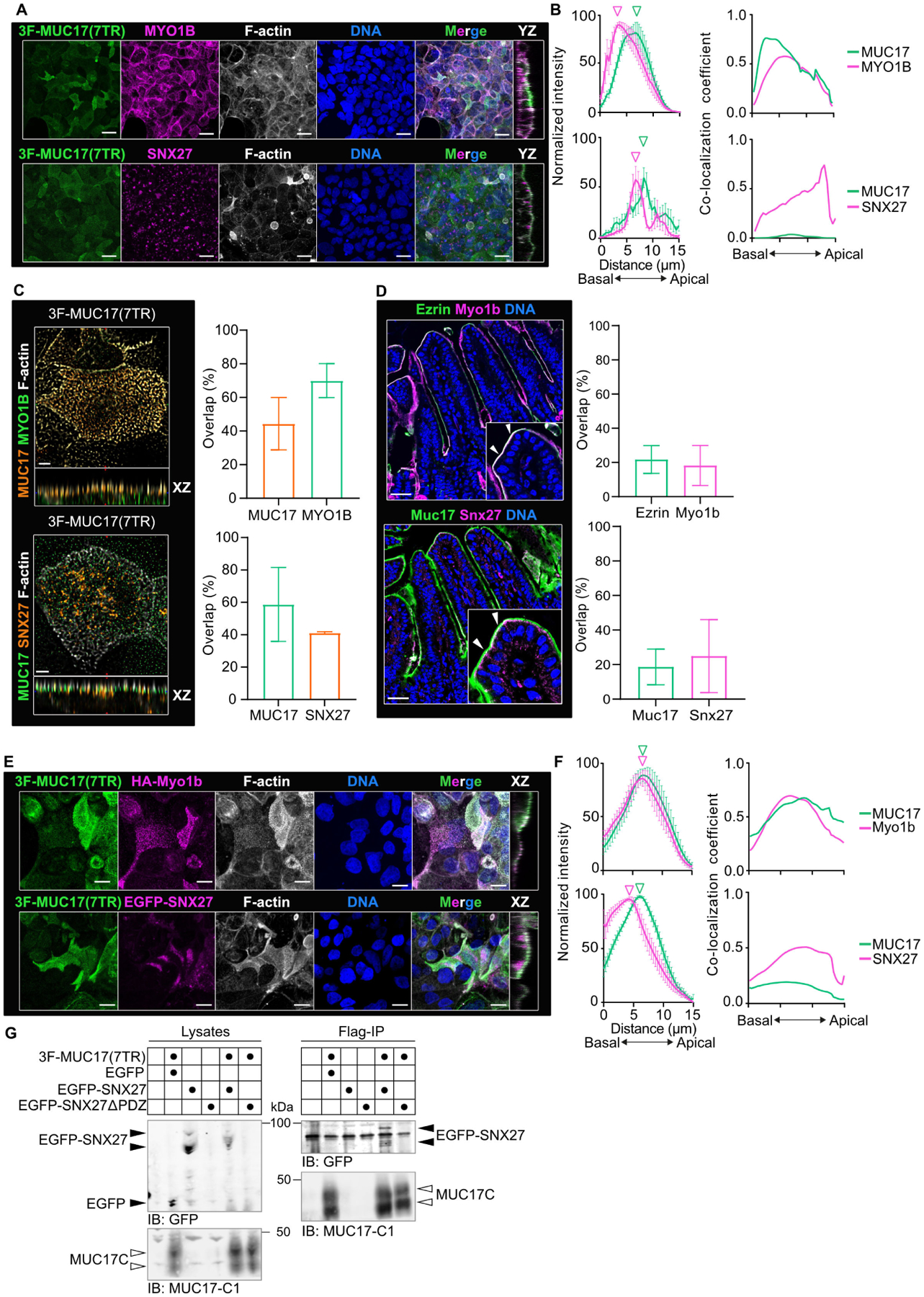
SNX27 interacts with membrane mucin 3F-MUC17(7TR). (**A**) Confocal images of 3F-MUC17(7TR) together with SNX27 or MYO1B, co-stained for F-actin and nuclear DNA in WT Caco-2 cells at 14 dpc. YZ view represents the orthogonal view of the XY maximum projections. Scale bar 20 μm. (**B**) Intensity profiles of 3F-MUC17(7TR) distribution in relation to MYO1B and SNX27 (left). The vertical arrowheads point to maximum intensity of each protein along the length of the cell; *n* = 3 scans and data are presented as mean ± SD. Fractional overlap expressed as co-localization coefficients of 3F-MUC17(7TR) with MYO1B or SNX27 and MYO1B or SNX27 with 3F-MUC17(7TR) in the upper and lower panel, respectively; *n* = 3 scans and data are presented as mean. (**C**) High-resolution Airyscan images of the brush border of WT Caco-2 cells stained for 3F-MUC17(7TR), MYO1B or SNX27, and F-actin at 14 dpc. XZ view represents the orthogonal view of the XY maximum projections. Scale bars 5 μm. Quantification of overlap between 3F-MUC17(7TR) and MYO1B and SNX27, respectively, in the brush border region (right); *n* = 3 scans. (**D**) Sections of mouse ileum stained for Ezrin, Myo1b, and nuclear DNA (top) and Muc17, Snx27, and DNA (bottom). The arrowheads in insets mark the brush border region. Scale bars 100 μm. Quantification of total overlap between Myo1b and Snx27 with either the microvillar marker Ezrin or Muc17, respectively (right); *n* = 3 scans. (**E**) Confocal images of 3F-MUC17(7TR) together with recombinant HA-Myo1b or EGFP-SNX27, co-stained for F-actin and nuclear DNA at 14 dpc. XZ view represents the orthogonal view of the XY maximum projections. Scale bars 5 μm. (**F**) Intensity profiles of 3F-MUC17(7TR) distribution in relation to HA-Myo1b and EGFP-SNX27, respectively (left). The vertical arrowheads point to maximum intensity of each protein along the length of the cell; *n* = 3 scans and data are presented as mean ± SD. Fractional overlap expressed as co-localization coefficients between 3F-MUC17(7TR) with HA-Myo1b or EGFP-SNX27 and HA-Myo1b or EGFP-SNX27 with 3F-MUC17(7TR) in the upper and lower panel, respectively; *n* = 3 scans and data are presented as mean. (**G**) A representative immunoblot of co-immunoprecipitations in HEK 293 cells expressing 3F-MUC17(7TR) and either EGFP-SNX27 or EGFP-SNX27ΔPDZ. Lysates represent 2% of the total cell lysate. Forty percent of the eluates were loaded on the gel. The solid arrowheads point to EGFP or isoforms of EGF-SNX27 and EGFP-SNX27ΔPDZ. The open arrowheads point to the C-terminus of 3F-MUC17(7TR)

Based on our interactome discovery, we first directed our attention to MYO1B and SNX27. MYO1B regulates the apical targeting of membrane proteins to the brush border [26], whereas SNX27 is involved in the recycling of membrane proteins through interactions with its N-terminal PDZ domain [27]. In Caco-2–3F-MUC17(7TR) cells, endogenous MYO1B localized to the subapical terminal web region and overlapped with 3F-MUC17(7TR) along the entire length of the microvilli (Figure 3A and B), as evident at higher magnification (Figure 3C). SNX27 was primarily restricted to discrete puncta within the terminal web region (Figure 3A and B), with a minor overlap with 3F-MUC17(7TR) in the microvilli (Figure 3C). Observations in differentiated Caco-2 cells were validated in the mouse ileum, where Myo1b overlapped with endogenous Ezrin in the apical brush border and Snx27 localized mainly to the terminal web below Muc17 in the brush border (Figure 3D). For additional validation, we defined the localization of recombinantly tagged rat Myo1b and human SNX27 in relation to 3F-MUC17(7TR) in differentiated Caco-2 cells. In line with the endogenous proteins in cultured cells and murine ileum, HA-Myo1b localized with 3F-MUC17(7TR) to the brush border of Caco-2 cells, whereas recombinant EGFP-SNX27 formed puncta below the brush border (Figure 3E and F, Figure S1A–B). MUC17 contains a conserved C-terminal class I PBM that can potentially mediate a PDZ interaction with SNX27 [6,28]. Therefore, we introduced the tagged constructs into HEK293 cells (Figure S1A–B) and used co-immunoprecipitation assays to investigate whether HA-Myo1b and EGFP-SNX27 interact with 3F-MUC17(7TR). EGFP-SNX27 coprecipitated with 3F-MUC17(7TR) (Figure 3G); however, due to the poor expression of EGFP-SNX27ΔPDZ, we did not observe an interaction between 3F-MUC17(7TR) and EGFP-SNX27ΔPDZ. Furthermore, we were not able to detect a direct interaction between HA-Myo1b and 3F-MUC17(7TR), suggesting that their association is either indirect or too weak to capture (data not shown).

**Figure 4:**
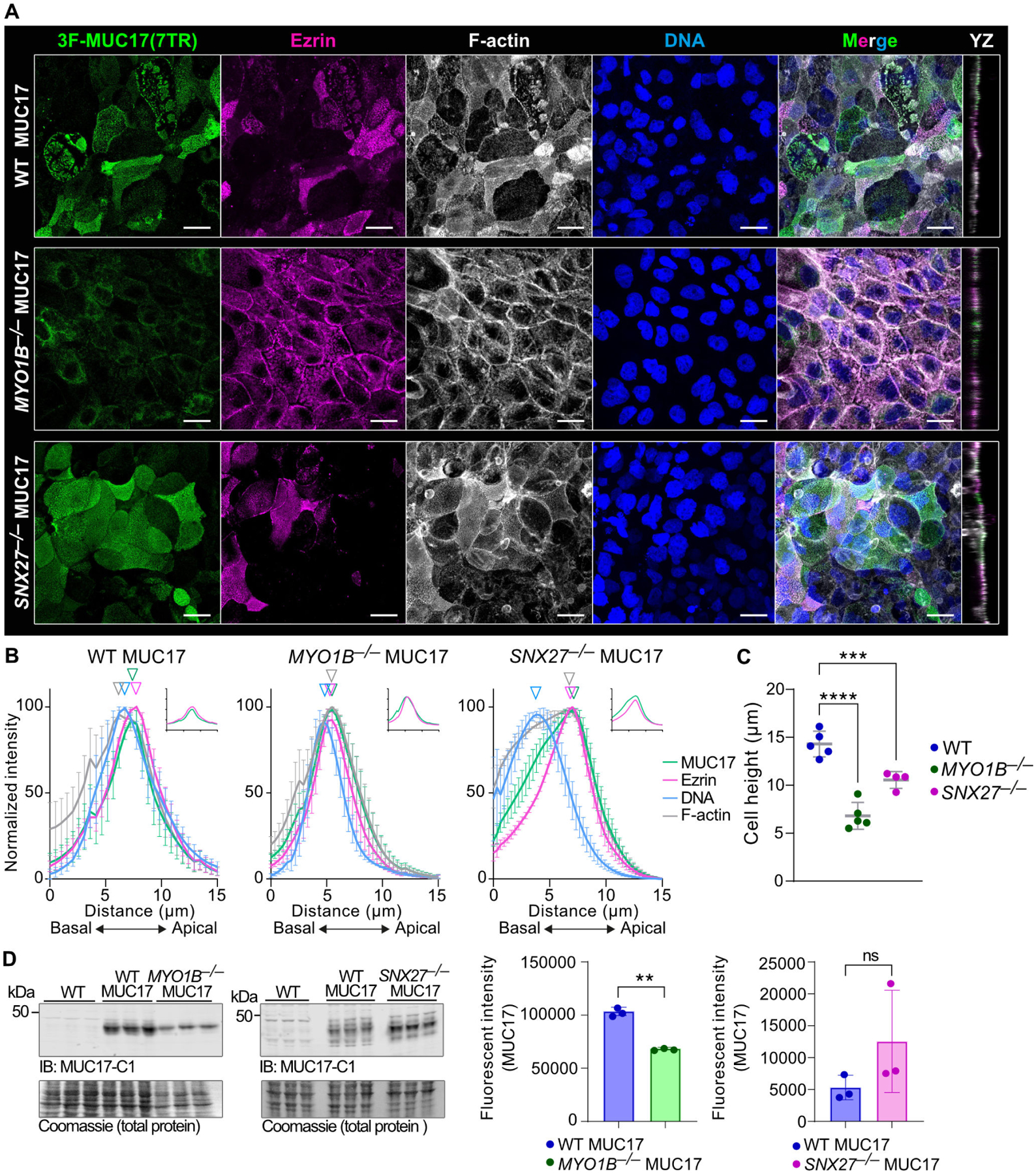
MYO1B regulates total protein amounts of 3F-MUC17(7TR). (**A**) Confocal images of the brush border region WT 3F-MUC17(7TR) and *MYO1B*^*−/−*^ 3F-MUC17(7TR), and *SNX27*^*−/−*^ 3F-MUC17(7TR) Caco-2 cells at 14 dpc, stained for 3F-MUC17(7TR), Ezrin, F-actin, and DNA. YZ view represents the orthogonal view of the XY maximum projections. Scale bars 20 μm. (**B**) Normalized intensity profiles of 3F-MUC17(7TR) distribution in relation to Ezrin and F-actin in WT and mutant Caco-2 cell lines. The vertical arrowheads point to maximum intensity of each protein along the length of the cell; *n* = 3 scans and 76 cells per cell line. Data are presented as mean ± SD. Absolute intensities of MUC17 (green) and Ezrin (magenta) are shown in insets. (**C**) Cellular height (in μm) of 3F-MUC17(7TR)-expressing WT, *MYO1B*^*−/−*^ and *SNX27*^*−/−*^ Caco-2 cells; *n* = 4–5 scans. Data are presented as mean ± SD. ***P = 0.0004, ****P < 0.0001 as determined by one-way ANOVA with Dunnett’s multiple comparisons test. (**D**) Determination of 3F-MUC17(7TR) protein levels in WT Caco-2 cells and cells lacking either MYO1B or SNX27 at 14 dpc (left panel). Fifteen percent of the total cell lysate was loaded on the gel for each cell line. The right panel represents a quantitative analysis of protein levels in WT and mutant Caco-2 cells normalized to the total amount of protein (Coomassie); *n* = 3 for each group. Data are presented as mean ± SD. **P < 0.01 as determined by unpaired t-test with Welch’s correction.

Based on the distinct apical and subapical localization of MYO1B and SNX27, we hypothesized that the two proteins regulate the apical targeting of 3F-MUC17(7TR). For that reason, we deleted MYO1B and SNX27 separately in Caco-2 cells and re-introduced 3F-MUC17(7TR) (Figure S2A–C). 3F-MUC17(7TR) remained in the apical brush border in *SNX27*^*−/−−/−*^ 3F-MUC17(7TR) cells, whereas apical 3F-MUC17(7TR) staining was largely lost in *MYO1B*^*−/−*^ 3F-MUC17(7TR) cells (Figure 4A and B). The staining pattern of microvillar ezrin was altered in *MYO1B*^*−/−*^ cells compared with WT and *SNX27*^*−/−*^ cells. Notably, total cellular height was significantly reduced in both *MYO1B*^*−/−*^ and *SNX27*^*−/−*^ cells expressing 3F-MUC17(7TR) (Figure 4C). An assessment of total protein levels showed a reduction of the total amounts of 3F-MUC17(7TR) in *MYO1B*^*−/−*^ cells (Figure 4D), suggesting that MYO1B does not directly impact the localization of 3F-MUC17(7TR) at the apical brush border but rather impacts total protein levels of the mucin. In conclusion, we demonstrated that both MYO1B and SNX27 localize to the brush border together with endogenous and recombinant MUC17 and that SNX27 interacts directly with recombinant MUC17.

### MYO5B regulates polarized MUC17 trafficking to the plasma membrane

**Figure 5:**
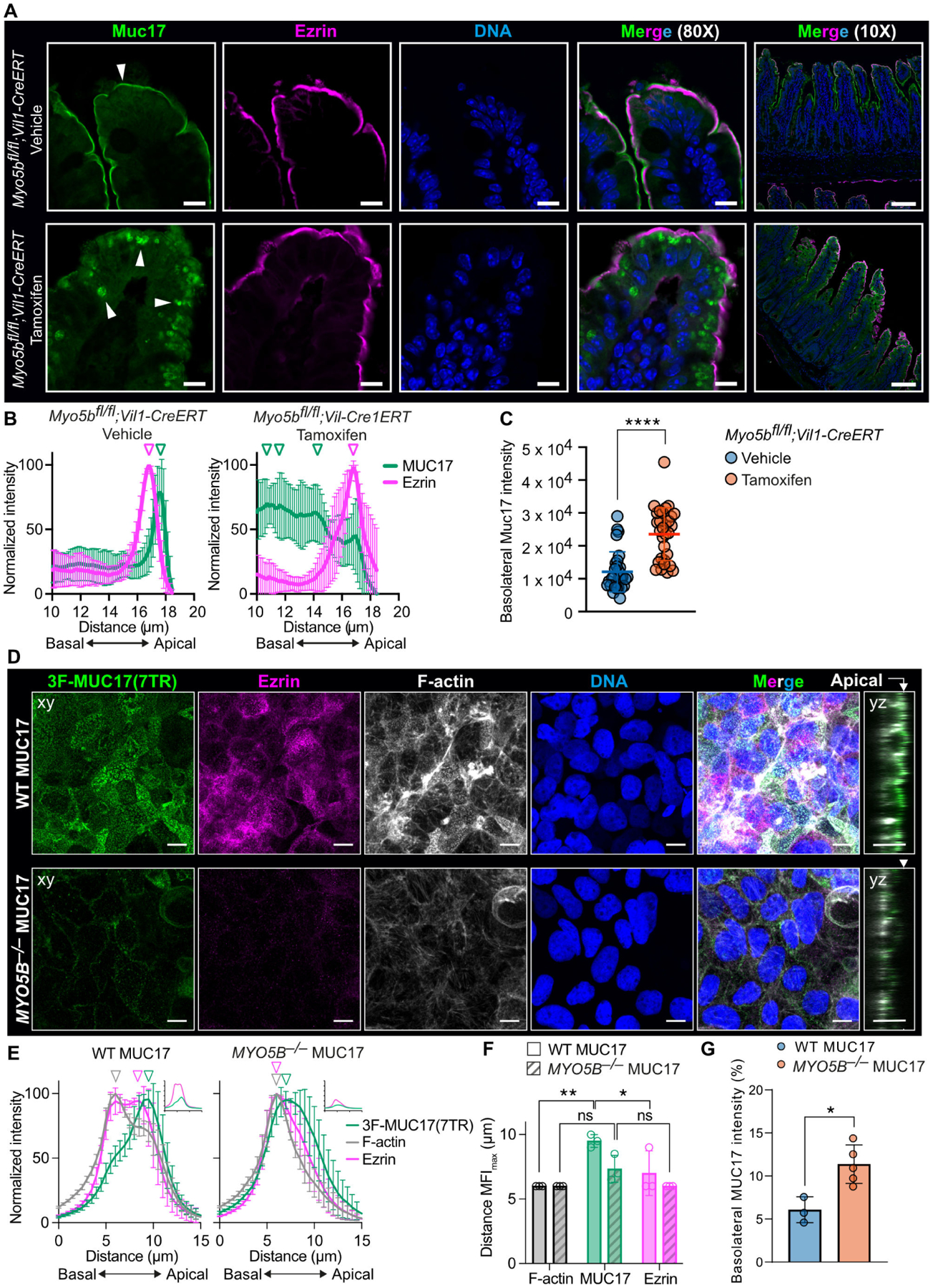
Endogenous and recombinant MUC17 reside intracellularly in enterocytes carrying a MYO5B deletion. (**A**) Ileal sections from *Myo5b*^*fl/fl*^*;Vil1-CreERT* mice, injected with vehicle or tamoxifen, stained for endogenous Muc17, Ezrin, and nuclear DNA. Arrowheads point to endogenous Muc17. Scale bar 100 μm and 10 μm in insets. (**B**) Intensity profiles of endogenous Muc17 and Ezrin in brush border regions in A. The vertical arrowheads point to maximum intensity of each protein along the length of the cell; *n* = 9 in each group. Data are presented as mean ± SD. (**C**) Quantification of the basolateral localization, defined as integrated fluorescence density, of endogenous Muc17 in *Myo5b*^*fl/fl*^*;Vil1-CreERT* mice, injected with vehicle or tamoxifen; *n* = 30 in each group. Data are presented as mean ± SD and analyzed by unpaired t-test. **** *P* < 0.0001. (**D**) Confocal images of WT and *MYO5B*^*−/−*^Caco-2 cells stained for 3F-MUC17(7TR), Ezrin, F-actin, and nuclear DNA at 14 days post-confluence. YZ view represents the orthogonal view of the XY maximum projections. The white arrow marks the apical membrane. Scale bars 20 μm. (**E**) Normalized intensity profiles of 3F-MUC17(7TR) distribution in WT and *MYO5B*^*−/−*^ Caco-2 cells in relation to Ezrin in D. The vertical arrowheads point to maximum intensity of each protein along the length of the cell; *n* = 3 in each group. Data are presented as mean ± SD. Absolute intensities of MUC17 (green) and Ezrin (magenta) are shown in insets for every intensity profile. (**F**) Semi-quantitative analysis of intensity profiles in E, showing the distance (μm) of the mean fluorescent intensity maxima of 3F-MUC17(7TR), Ezrin and F-actin in WT and *MYO5B*^*−/−*^ Caco-2 cells. Data are presented as mean ± SD and analyzed by two-way ANOVA corrected for multiple comparisons using Sidak. *P < 0.05 and **P < 0.01. (**G**) Quantification of the basolateral localization of 3F-MUC17(7TR) in WT and *MYO5B*^*−/−*^ Caco-2 cells; *n* = 6 in each group. Data are presented as mean ± SD and analyzed by unpaired t-test. *P < 0.05.

Since we discovered the monomeric MYO1B in the interactome of 3F-MUC17(7TR), we asked whether other non-muscle myosins were associated with MUC17. Mining of public single-cell RNA-sequencing data sets revealed that *MYO1A*, *MYO5B*, and *MYO7B* transcripts are highly enriched in enterocytes (Figure S2D). MYO5B is particularly interesting since it transports Rab8^+^Rab11^+^ endosomes carrying membrane proteins to the apical brush border and regulates cell polarity [29,30]. Moreover, loss-of-function mutations in the *MYO5B* gene lead to Microvillus inclusion disease (MVID) in humans [31]. To determine the impact of MYO5B on MUC17 trafficking, we stained for endogenous Muc17 and Ezrin in ileal sections of *Myo5b*^*fl/fl*^*;Vil1-CreERT* mice injected with vehicle or tamoxifen to induce deletion of the *Myo5b* gene in *Vil1*-expressing intestinal epithelial cells. In *Myo5b*^*fl/fl*^*;Vil1-CreERT* mice injected with tamoxifen, MUC17 was completely absent from the brush border and restricted to large intracellular vesicles, while Ezrin was retained in the brush border (Figure 5A and B). MUC17 was mislocalized to the basolateral membrane in mice lacking Myo5b (Figure 5C). To further investigate how MYO5B regulates MUC17 trafficking, we deleted *MYO5B* in Caco-2–3F-MUC17(7TR) cells (Figure S2A, E). While 3F-MUC17(7TR) resided in the apical brush border of WT Caco-2 cells, apical 3F-MUC17(7TR) staining was largely lost in *MYO5B*^*−/−*^ cells, thereby reproducing the phenotype observed in mice carrying a *Myo5b* deletion (Figure 5D and E). Moreover, the cellular height of *MYO5B*^*−/−*^ cells was significantly reduced (Figure S2F), and the cells demonstrated a dramatic reduction in microvillus-associated Ezrin, which has been observed in previous reports [32]. In contrast to 3F-MUC17(7TR), which localized to the outer tip of Ezrin-stained microvilli in WT cells, a small fraction of apical 3F-MUC17(7TR) observed in *MYO5B* ^−/−^ cells was redistributed to the entire length of microvilli (Figure 5E and F). The remaining fraction of 3F-MUC17(7TR) is localized to the basolateral membrane (Figure 5G).

**Figure 6:**
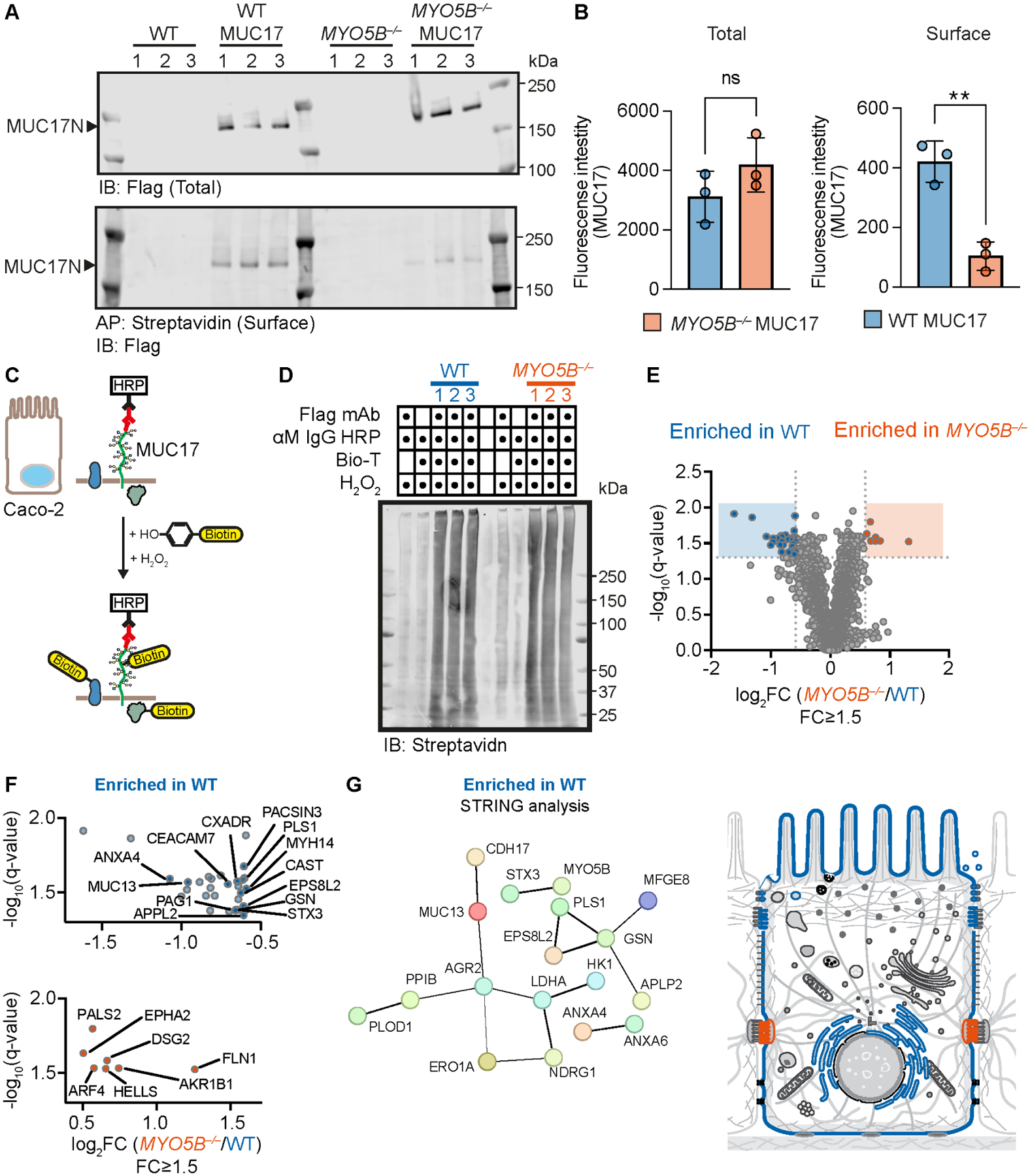
MYO5B regulates 3F-MUC17(7TR) trafficking to the plasma membrane. (**A**) Immunoblots showing streptavidin affinity purification of surface biotinylated 3F-MUC17(7TR) in WT and *MYO5B*^*−/−*^Caco-2 cells at 14 days post-confluence. (**B**) Semi-quantitative analysis of band densities in A for total and surface biotinylated 3F-MUC17(7TR); *n* = 3 for each group. Data are presented as mean ± SD. **P < 0.01 as determined by unpaired t-test with Welch’s correction, assuming non-equal SD. (**C**) Schematic illustration of biotin proximity labeling assay. HRP, horseradish peroxidase. (**D**) Immunoblot of eluates from biotin proximity labeling experiments of WT 3F-MUC17(7TR) and *MYO5B*^*−/−*^ 3F-MUC17(7TR) Caco-2 cells. (**E**) Volcano plot of proteins enriched in WT 3F-MUC17(7TR) and *MYO5B*^*−/−*^ 3F-MUC17(7TR) Caco-2 cells from the biotin proximity labeling experiment; *n* = 3 for each group. Proteins with significantly different abundance (fold change ≥ 1.5, padj < 0.05) are highlighted in blue for WT and red for 3F-MUC17(7TR)-expressing cells. (**F**) Significantly enriched proteins for WT 3F-MUC17(7TR) (blue) and *MYO5B*^*−/−*^ 3F-MUC17(7TR) (red) Caco-2 cells. Detailed visualization of differentially enriched proteins shown in E. (**G**) Visualization by STRING analysis of the functional protein network proximal to 3F-MUC17(7TR) in WT Caco-2 cells (blue) and the subcellular localization of the proximal proteome of 3F-MUC17(7TR) in WT (blue) and *MYO5B*^*−/−*^ Caco-2 cells (red).

Due to the dramatic redistribution of 3F-MUC17(7TR) in *MYO5B*^*−/−*^ cells, we sought to investigate if the loss of MYO5B affects the surface pool of 3F-MUC17(7TR) by applying biotin surface labeling followed by streptavidin affinity purification (Figure 6A). While the deletion of *MYO5B* did not impact the total protein levels of 3F-MUC17(7TR), *MYO5B*^*−/−*^ cells presented significantly less 3F-MUC17(7TR) on the apical surface compared to the WT cells (Figure 6B), thus providing additional evidence for the loss of apical 3F-MUC17(7TR) localization upon deletion of MYO5B. Next, we took advantage of biotin proximity labeling by antibody recognition coupled with quantitative mass spectrometry to obtain a quantitative comparison of the intracellular context surrounding 3F-MUC17(7TR) in WT and *MYO5B*^*−/−*^ Caco-2 cells (Figure 6C–E, Table S2). The proximal proteome of 3F-MUC17(7TR) in WT cells provided a unique insight into the protein environment that 3F-MUC17(7TR) encounters during intracellular trafficking to the brush border. In WT cells, the identified proteins proximal to 3F-MUC17(7TR) were highly enriched in the gastrointestinal tract and participated in vesicle trafficking (ANX4, PACSIN3, APPL2, STX3) and cytoskeleton remodeling (MYH14, PLS1, EPS8L2, GSN) (upper panel Figure 6F and G, Table S2). STX3 is particularly important since it participates in membrane fusion of endosomes transported by MYO5B, and mutations in STX3 are associated with variants of MVID [31], indicating the MUC17 encounters multiple components of MYO5B-mediated trafficking. In contrast with the WT cells, the proximal proteome of 3F-MUC17(7TR) in *MYO5B*^*−/−*^ cells was dominated by proteins associated with basolateral membrane domains (lower panel Figure 6F and G, Table S2). We conclude that MYO5B regulates the apical targeting of endogenous and recombinant MUC17 to the enterocytic brush border.

### MYO1B and SNX27 regulate the slow turnover of recombinant MUC17 in the membrane

**Figure 7:**
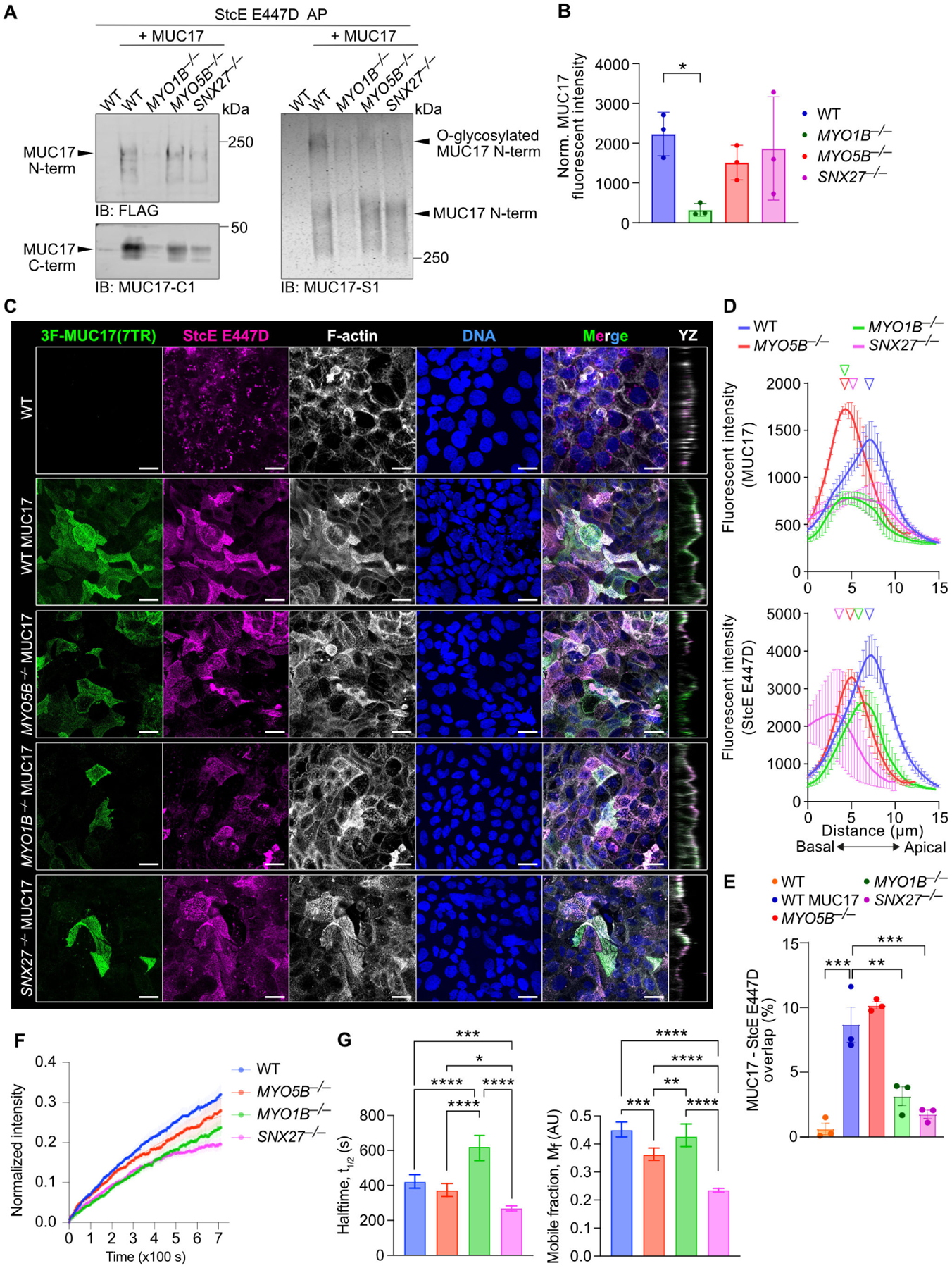
Reduced apical 3F-MUC17(7TR) targeting in MYO1B-deficient cells. (**A**) Assessment of the maturation of 3F-MUC17(7TR) by affinity purification (AP) using immobilized StcE E447D in WT and mutant Caco-2 cells. Eluates (40%) were analyzed by immunoblotting (IB) (left panel) or in-gel western (right panel) using Flag, MUC17-C1 or MUC17-S1 antibodies. (**B**) Densitometric analysis of total 3F-MUC17(7TR) normalized to total protein in each sample used for StcE E447D AP in A; *n* = 3 for each group. Data are presented as mean ± SD *P < 0.05 as determined by Kruskal-Wallis and Dunn’s multiple comparisons test. (**C**) Confocal images of WT, *MYO5B*^*−/−*^, *MYO1B*^*−/−*^, and *SNX27*^*−/−*^ cells at 14 days post-confluence stained for 3F-MUC17(7TR) and fluorescently conjugated StcE E447D, alongside YZ orthogonal views of the maximum projections. Scale bars 20 μm. (**D**) Absolute intensity profiles of 3F-MUC17(7TR) (upper panel) and StcE E447D (lower panel) for WT and mutant Caco-2 cells in C. The vertical arrowheads point to maximum intensity of each protein along the length of the cell; *n* = 3 scans and a mean of 82 cells per cell line. (**E**) Proportion of 3F-MUC17(7TR) signal intensity that overlaps with StcE E447D in C; *n* = 3 for each group. Data are presented as mean ± SD and analyzed by two-way ANOVA corrected for multiple comparisons using Sidak, *P < 0.05, **P < 0.01, ***P < 0.001 . (**F**) Curves representing recovery after photobleaching of 3F-MUC17(7TR)-bound StcE E447D in the plasma membrane of WT and mutant Caco-2 cells. Data are represented as mean ± SEM; *n* = 19–23 per group. (**G**) Halftime and mobile fraction of StcE E447D-labeled 3F-MUC17(7TR) in WT and mutant cells, extracted from F; *n* = 19–23 per group. Data are represented as mean ± SD and analyzed by one-way ANOVA corrected for multiple comparisons using Tukey, *P < 0.05, **P < 0.01, ***P < 0.001, and ****P < 0.0001.

Our assessment of 3F-MUC17(7TR) localization in fixed differentiated Caco-2 cells revealed that the deletion of MYO5B had a dramatic impact on the targeting of 3F-MUC17(7TR) to the apical brush border, while the influence of MYO1B and SNX27 was more modest. Therefore, we asked whether MYO1B, MYO5B, and SNX27 affected the kinetics of 3F-MUC17(7TR) at the brush border in live cells. To label surface-exposed 3F-MUC17(7TR) in live cells, we took advantage of the inactive E447D mutant of StcE that only binds *O*-glycosylated mucin domains. StcE E447D immobilized on cyanogen bromide (CNBr)-activated sepharose was able to pull down mature *O*-glycosylated 3F-MUC17(7TR) from lysates of Caco-2 cells (Figure 7A, Figure S3). StcE E447D also captured mature 3F-MUC17(7TR) expressed in *MYO5B*^*−/−*^ and *SNX27*^*−/−*^ cells and to a lower extent in *MYO1B*^*−/−*^ cells due to reduced overall 3F-MUC17(7TR) expression (Figure 7B). This data indicates that 3F-MUC17(7TR) *O*-glycosylation in the Golgi apparatus was not affected by the deletion of the individual trafficking proteins. Next, we used imaging to evaluate if fluorescently labeled StcE E447D detected surface-exposed 3F-MUC17(7TR) in Caco-2 monolayers. While StcE E447D staining in permeabilized non-transfected WT cells was mainly intracellular, there was a strong overlap between StcE E447D and 3F-MUC17(7TR) in the apical brush border of transfected WT cells (Figure 7C–E). In cells lacking MYO5B, the amount of apical E447D StcE staining was lower than in WT cells, while the remaining small surface population of 3F-MUC17(7TR) was stained with StcE E447D (Figure 7C–E). In *MYO1B*^*−/−*^ and *SNX27*^*−/−*^ cells, we observed a significant reduction of the overlap between 3F-MUC17(7TR) and StcE E447D in the brush border (Figure 7C–E). Thus, immunofluorescence indicated that StcE E447D serves as an appropriate probe for monitoring surface-exposed recombinant MUC17 in live cells. Consequently, we took advantage of fluorescence recovery after photobleaching (FRAP) to measure the kinetics of 3F-MUC17(7TR) at the brush border in live cells. Fluorescent recovery of 3F-MUC17(7TR) in WT cells was slow (t_1/2_ = 6 minutes) and incomplete at the end point of the experiment (11.7 minutes) (Figure 7F and G, left panel). The mobile fraction, representing the lateral diffusion of 3F-MUC17(7TR) within the plasma membrane, was 45% (Figure 7G, right panel). Hence, under baseline conditions, 3F-MUC17(7TR) is a stable transmembrane glycoprotein with a low lateral diffusion rate within the membrane and a low exchange rate between the membrane and the cytoplasm. Compared with WT cells, the recovery rate of 3F-MUC17(7TR) was significantly slower in *MYO1B*^*−/−*^ cells (t_1/2_ = 10 minutes) and only modestly faster in *MYO5B*^*−/−*^ and *SNX27*^*−/−*^ cells. The mobile fraction of 3F-MUC17(7TR) decreased by 50% in *SNX27*^*−/−*^ cells, suggesting that SNX27 participates in stabilizing 3F-MUC17(7TR) at the brush border. Subtle differences in the mobile fraction of 3F-MUC17(7TR) were observed in *MYO1B*^*−/−*^ and *MYO5B*^*−/−*^ cells. Together, our results indicate that MYO1B regulates the turnover of 3F-MUC17(7TR) at apical brush border via membrane-cytoplasmic exchange, whereas SNX27 influences the stability of the membrane mucin by decreasing stabilizing interactions and enhancing lateral diffusion within the brush border membrane. Importantly, MYO5B regulates the targeting of 3F-MUC17(7TR) to the plasma membrane without influencing MUC17 dynamics once it reaches the apical membrane.

## Discussion

We have previously identified a MUC17-based glycocalyx covering the surface of enterocytes. Importantly, the absence of the enterocytic glycocalyx results in increased bacterial contact with the enterocytic brush border [5], highlighting the importance of a precise regulation of MUC17 targeting in enterocytes. In this study, we mapped the intracellular pathways responsible for apical targeting of a recombinant MUC17 with seven tandem repeats in the epithelial cell line Caco-2. Based on our findings, we present the molecular processes underlying the apical targeting of MUC17 in a proposed model, as illustrated in Figure S4.

Confluent Caco-2 cells undergo a differentiation process, mirroring the *in vivo* maturation of enterocytes along the crypt-villus axis. Differentiated Caco-2 cells are characterized by a less pronounced tumorigenic phenotype, cytoskeletal rearrangements [22], and tightly packed microvilli held together by tip links formed by an IMAC [24]. Our investigation showed that the recombinant MUC17 localized to the plasma membrane regardless of the differentiation status of Caco-2 cells, whereas the density of the glycocalyx increases with microvillus packing as cells differentiate. Our findings are consistent with the apical localization of endogenous MUC17 in differentiated enterocytes lining villi in the human and mouse ileum [5] and suggest that MUC17 insertion in the apical membrane precedes microvillus assembly and packing. As enterocytes reach a differentiated state marked by an organized brush border, we postulate that the tight packing of microvilli positions MUC17 at the distal tip of individual microvillus protrusions, where a dense glycocalyx is established.

PDZ proteins regulate membrane mucin trafficking through the Golgi apparatus and retention at the apical cell membrane [6,33], but we lack a comprehensive understanding of how large membrane mucins such as MUC17 are targeted to the plasma membrane in enterocytes. Here, we developed enrichment protocols that identified primarily cytoplasmic proteins involved in the recycling, processing, and transport of a recombinant MUC17. PDZ-domain containing SNX27 directs endocytosed proteins from the early endosomes to the plasma membrane [34]. We showed that recombinant MUC17 interacts with SNX27. Given the subapical positioning of SNX27 in enterocytes, our findings suggest that SNX27 directs MUC17 to the terminal web region before the membrane mucins is moved into the brush border compartment. We also showed that SNX27 affects the stability of the recombinant MUC17 at the apical brush border. In *SNX27*^*−/−*^ cells, a higher proportion of recombinant MUC17 remained static within the brush border, suggesting that SNX27 decouples MUC17 from tethering complexes at the base of microvilli.

The role of unconventional non-muscle myosins in microvillar assembly and function has been extensively characterized [35]. Our mapping of the MUC17 interactome identified the monomeric myosin MYO1B, which is known to regulate the targeting of amino acid transporters to the brush border of kidney cells [26]. MYO1B interacts with actin filaments but cannot transport vesicular cargo along filaments [36,37]. Actin-bound MYO1B induces tubule formation in endosomal and lysosomal membranes, as well as the trans-Golgi network, thereby controlling protein trafficking between endocytic compartments [38,39]. Both endogenous and recombinant MYO1B localized with MUC17 at the brush border of cultured epithelial cells and enterocytes in the mouse Ileum. Depletion of MYO1B resulted in reduced levels of recombinant MUC17 and slower turnover in the brush border. The latter phenotype could be explained by a higher abundance of microvillar clusters that restrict the diffusion of recombinant MUC17 in the brush border membrane of *MYO1B*^*−/−*^ cells.

We also addressed the role of the dimeric unconventional myosin MYO5B in the targeting of MUC17 to the apical membrane. Specific transporters such as NHE3, AQP7, and SGLT1, but not cystic fibrosis transmembrane conductance regulator (CFTR), require MYO5B for their correct targeting to the apical brush border [40] and mutations in the *MYO5B* gene have been linked to the congenital diarrheal disorder MVID caused by the mislocalization of membrane transporters that maintain cell and fluid homeostasis [41]. The deletion of *Myo5b* in the mouse small intestine or *MYO5B* in Caco-2 cells resulted in a dramatic loss of both endogenous and recombinant MUC17 from the brush border, and proximity labeling revealed that the recycling defects associated with *MYO5B* deletion abolished the polarized targeting of recombinant MUC17 to the apical membrane. Our findings are in line with previous investigations of the role of MYO5B in regulating apical cell polarity [42]. Since not all apically positioned proteins are regulated by MYO5B, our work identified MUC17 as a new apical cargo for this critical myosin.

In conclusion, we show that MYO1B, MYO5B, and SNX27 regulate the apical targeting of the glycocalyx-forming membrane mucin MUC17 in enterocytes. Unraveling the cellular mechanisms that govern the formation of the glycocalyx barrier sheds light on fundamental cellular processes for combatting bacterial encroachment on the intestinal epithelium. Importantly, our insights into the targeting of MUC17 could prove critical for identifying molecular defects that render enterocytes sensitive to bacterial invasion (Table 1).

## Materials and methods

### Plasmids

Copy DNA (cDNA)-encoding recombinant MUC17(7TR) with an N-terminal 3xFlag tag was generated using Gibson Assembly (E2611S, NEB) following the manufacturer’s protocol. A cDNA insert with the endogenous MUC17 signal sequence fused to 3xFlag followed by sequence-optimized last 7 N-terminal tandem repeats of the mucin was prepared (see Figure S5). The insert was equipped with 5′ and 3′ flanking sequences overlapping with the pXL-CAG-Zeocin-3xF2A plasmid [43] digested with NotI and AscI restriction enzymes. Rat Myo1b (Plasmid #135064, Addgene) was cloned into pXL-CAG-Zeocin-3xF2A replacing the Myc-tag with an N-terminal HA-tag by introducing a stop codon using a two-step PCR approach and primer pair 5′-CGCCACCATGTACCCATACGATGTTCCAGATTACGCTGCCAAGAAG GA-GGTAAAATCCTC-3′ and 5′-TCACC-GAGAATTCAGCCGTGG-3′ followed by primer pair 5′-CA GGGCCAGATATCGCCGCCACCATGT-ACCCATACGATGT-3′ and 5′-TGAGCTTTTGC-TCTGGTCA CCGAGAATTCAGCC-GTGGGACA-3′. SNX27 and SNX27ΔPDZ (a kind gift by Prof. Peter J. Cullen [28,34]) with N-terminal Enhanced Green Fluorescent Protein (EGFP) tags were cloned into the pXL-CAG-Zeocin-3xF2A using primer pair 5′-CAGGGCCAGATATCGCCGCCACCATGGTGAGC-AAGG-3′ and 5′-TGAGCTTTTGCTCTGGCTAATATTCCTCTTTTCTCCACTTGAGCT-3′. The following guide RNAs were used for the deletion of *MYO1B*, *MYO5B*, and *SNX27* genes using the pLentiCRISPR v2 vector according to the protocol by the Zhang lab [44,45] *MYO1B* 5′-ATGAAGGTCTCCTCATTGAG-3′, *MYO5B* 5′-GCGCTCAGCTGAGTTAACCA-3′, and *SNX27* 5′-GCTACGGCTTCAACGTGCG-3′. Vectors containing gRNAs were transformed into One Shot^™^ Stbl3^™^ Chemically Competent *Escherichia coli* according to the manufacturer’s protocol (Invitrogen) and confirmed by sequencing using primer U6-F GA GGGCCTATTTCCCATGATT.

### Immunohistological sections of the mouse ileum

Wild-type C57BL/6 N mice were maintained under standardized conditions of temperature (21–22°C) and illumination (12-hour light/dark cycle) with food and water ad libitum. The Swedish Laboratory Animal Ethical Committee in Gothenburg approved the experiments conducted in this study (ethical permit 2285–19). The care and use of animals were performed in accordance with the Swedish animal welfare legislation, which meets the European Convention for the Protection of Vertebrate Animals used for Experimental and other Scientific Purposes (Council of Europe N° 123, Strasbourg 1985) and the European Union Directive 2010/63/EU on the protection of animals used for scientific purposes. Animals were anesthetized with isoflurane followed by cervical dislocation. Animals of 6–8 weeks of age and both sexes were used. Weaning occurred on day 21 after birth. For investigation of MYO5b function in mouse ileum, Cre recombinase was activated in 8- to 10-week-old *VillinCreErt2;Myo5b*^*fl/fl*^ by one intraperitoneal injection of tamoxifen (2 mg). Tamoxifen-injected *Myo5b*^*fl/fl*^ mice and *VillinCreErt2;Myo5b*^*fl/+l*^ mice were used as controls [29]. All *VillinCreErt2;Myo5b*^*fl/fl*^ and control mice were killed four days after the tamoxifen dose. The care, maintenance, and treatment of *VillinCreErt2;Myo5b*^*fl/fl*^ mice followed protocols approved by the Institutional Animal Care and Use Committee of Vanderbilt University.

### Cell culture, transfections, and CRISPR/Cas9-mediated gene deletion

Caco-2 (ATCC HTB-37) and HEK293T (ATCC CRL-1573) cells were cultured in Iscove’s modified Dulbecco’s medium (IMDM, Invitrogen Life Technologies, Carlsbad, CA) containing 10% (v/v) FBS at 37°C in 5% CO2. Caco-2 cells for SILAC (#A33969, #88,210 Thermo Fisher Scientific) were cultured in Dulbecco’s modified Eagle medium supplemented with ^13^C_6_ L-lysine and ^13^C_6_L-arginine (heavy medium) or ^12^C_6_L-lysine and ^12^C_6_L-arginine (light medium), respectively, for five passages to ensure complete incorporation (> 95%). Transfections to generate stable clones were performed with 50% and 80% confluent Caco-2 and HEK293T cells, respectively, seeded in 9.6-mm^2^ wells. All transfections to introduce recombinant constructs were performed using Lipofectamine^®^ 2000 (11668019, Thermo Fisher Scientific) and the PiggyBac transposon system with a transposase (pCAG-mPB-orf):transposon (pXL-BacII-CAG-Zeocin-triple-F2A) ratio of 1:2.5. Transfected cells were incubated with a total of 4 μg DNA and 10 μl of Lipofectamine^®^ 2000 complex for 72 hours and selected for another 14 days with 300 μg/ml Zeocin. For selected mutant Caco-2 cell lines, cells were selected for three days with 700 μg/ml of G418 after transfection with CRISPR/Cas9 plasmids, and individual colonies were picked for expansion and screening by immunoblotting.

## Co-immunoprecipitation

### Method 1 with non-ionic IGEPAL detergent

3 × 10^5^ of 3F-MUC17(7TR) Caco-2 (heavy) and WT Caco-2 (light) cells were seeded into 9.6-mm^2^ wells and used for pull-down experiments at 14 dpc. Re-CLIP was performed by rinsing the cells three times with 37°C PBS followed by incubating cells with 1.25 mM DSP for 2 minutes at 37°C. DSP was quenched at room temperature (RT) with three washes of Tris-buffered saline (TBS). Non-cross-linked cells were washed three times in TBS at RT. Cells were subjected to Flag-immunoprecipitations as previously described [46]. Briefly, cells were washed 3 × 5 minutes in 37°C PBS, lysed in 1.5 μl ice-cold lysis buffer 1 (0.5 % IGEPAL, 250 mM NaCl, 50 mM Tris/HCl pH 7.4, 1 mM EDTA, 1X cOmplete EDTA-free protease inhibitor cocktail (11697498001, Roche), 1 mM phenylmethylsulfonyl fluoride(PMSF), 15 μl of phosphatase inhibitor cocktails 2 (P5726, Sigma-Aldrich) and 3 (P0044, Sigma-Aldrich)), and incubated at 4°C for 20 minutes on an orbital shaker. Cells were collected by scraping and sonicated for 3 minutes in a water-bath sonicator with ice at 10°C. The cell lysate was cleared by centrifugation at maximum speed for 30 minutes at 4°C, and 50 μl of the supernatant was saved as input. A 50 μl of EZview^™^ Red ANTI-FLAG^®^ M2 affinity gel (F2426, Sigma-Aldrich), equilibrated in lysis buffer 1, was added to the sample that was incubated overnight at 4°C on rotation. Beads were washed three times in lysis buffer 1 and three times in lysis buffer 1 without IGEPAL at 4°C. Enriched proteins were eluted by adding 25 μl 3xFLAG peptide (4 μg/μl) for 30 minutes at 4°C. Eluted proteins were separated from beads with Corning Costar Spin-X filter units (#CLS8162, Sigma-Aldrich) and stored at −20°C until further processing.

### Method 2 with ionic SDS detergent

Cells were cross-linked with 1.25 mM DSP for 2 minutes at 37°C followed by three washes in TBS. Cells were lysed in 500 μl of lysis buffer 2 (1% SDS, 250 mM NaCl, 50 mM Tris/HCl pH 7.4, 1 mM EDTA, 1× cOmplete EDTA-free protease inhibitor cocktail, 1 mM PMSF, 3 μM Beta-Glycerol phosphate, 15 μl phosphatase inhibitor cocktails 2 and 3). Cell lysates were sonicated for 10 seconds and centrifuged at maximum speed for 30 minutes at 4°C. Supernatants from heavy and light samples were mixed at a 1:1 ratio and diluted 1:10 to reach 0.1% SDS final concentration. Then, 50 μl of EZview^™^ Red ANTI-FLAG^®^ M2 affinity gel was added to each sample and incubated overnight at 4°C on rotation. Beads were washed three times in TBS + 0.1% SDS and eluted by the addition of 50 μl of elution buffer (1% SDS, 100 mM Tris pH 8.0, 100 mM DL-dithiothreitol (DTT)) and boiled at 95°C for 5 minutes. Eluted proteins were separated from beads with Costar Spin-X filter units (8160, Corning) and stored at −20°C until further processing.

### Co-immunoprecipitations in HEK293 cells

6 × 10^5^ HEK293T cells stably expressing recombinant constructs were seeded in 9.6-mm^2^ wells and used for pull-down experiments. Co-immunoprecipitations were performed in the absence of DSP according to method 1 with the additional step of blocking the EZview^™^ Red ANTI-FLAG^®^ M2 affinity gel with 5% BSA in TBS for 2 hours at 4°C on rotation and washed two times in lysis buffer 1 without IGEPAL before performing immunoprecipitation.

### Cell surface biotinylation

3 × 10^5^ Caco-2 cells were seeded on 9.6-mm^2^ wells and let to differentiate for 14 dpc. To label the surface proteins, cells were washed three times with ice-cold PBS and incubated with 0.25 μg/ml biotin hydrazide (66640–86–6, Sigma-Aldrich) in cold PBS for 1 hour on ice. After biotin labeling, cells were washed three times with TBS (pH 7.4) and left in the last TBS wash for 20 minutes at RT. Cells were lysed in 1 ml of lysis Buffer (1% Triton-X, 25 mM Tris/HCl pH 7.4, 150 mM NaCl, 1 mM EDTA, 4% glycerol supplemented with 1X cOmplete EDTA-free protease inhibitor cocktail) for 10 minutes on ice. Lysates were homogenized by sonication and centrifuged at 16,000 g for 30 minutes at 4°C. Next, 50 μl of cell lysate was mixed with a reducing sample buffer and used as an input loading control. The 30 μl of EZview^™^ Red Streptavidin Affinity Gel (E5529, Sigma-Aldrich) was added to each supernatant and incubated on rotation for 2 hours at 4°C. After three washes with lysis buffer, the bound material was eluted with reducing sample buffer and boiled at 95°C for 5 minutes. Samples were separated on a 4–15% gel by SDS-PAGE and transferred to PVDF-FL membranes (IPFL00010, Merck). Membranes were blocked with 5% non-fat milk in PBS and incubated with primary antibodies diluted in 5% non-fat milk in PBS + 0.1% Tween-20 (PBS-T) overnight at 4°C. After three PBS-T washes, protein bands were visualized with the Odyssey CLx imaging system (LI-COR Biosciences). Total biotinylated proteins in samples were detected with Alexa Fluor^™^ 790 Streptavidin Conjugate (1:20,000, S11378, Thermo Fisher Scientific). Band densities were quantified using Image Studio quantification software (LI-COR Biosciences).

### Expression and labeling of StcE and StcE E447D

Tuner (DE3) competent cells (70623, Sigma-Aldrich) were transformed with pET28b-StcE-Δ35-NHis or pET28b-StcE-E447D-Δ35-NHis (kind gift from Prof. Carolyn Bertozzi [47]) and grown on LB Agar with kanamycin at 37°C overnight. A single colony was pre-cultured in 10 ml LB kanamycin overnight, and the preculture expanded in 1 L LB kanamycin until an optical density of 0.85 was reached. Protein production was induced with 0.2 mM isopropyl β-D-thiogalactopyranoside (IPTG) at 30°C overnight. Bacterial cells were centrifuged at 3500 × g for 20 minutes at 4°C, resuspended in 20 ml of ice-cold PBS, and centrifuged at 3500 × g for 20 minutes at 4°C. Cell pellets were resuspended in 20 ml of ice-cold Binding Buffer (20 mM sodium phosphate, 300 mM NaCl, 20 mM imidazole, pH 7.4.) containing 2.5X Roche Complete EDTA-free protease inhibitor cocktail (11873580001, Sigma-Aldrich). The bacterial slurry was sonicated for 8 × 30 seconds at 50% duty in a water bath maintained at 4°C. Lysates were centrifuged at 22,000 × g at 4°C for 20 minutes and poured over 4 ml of HisPur Cobolt resin (89964, Thermo Fisher Scientific). The slurry was rotated at 4°C for 1 hour and spun down at 700 × g for 2 minutes. The resin was washed three times with Binding Buffer including a protease inhibitor cocktail, and the bound protein was eluted at 4°C with three subsequent 15-minute elutions using 3 ml of elution buffer (20 mM sodium phosphate, 300 mM NaCl, 500 mM imidazole, pH 7.4). Elution fractions were pooled and dialyzed against 5 L PBS at 4°C overnight, followed by a second round of dialysis in 5 L PBS for 4 hours at 4°C.

For enrichment of 3F-MUC17(7TR) using StcE E447D, StcE E447D was coupled to CNBr-Activated Sepharose 4B (17043001, Cytiva). One gram of CNBr-activated sepharose was resuspended in 10 ml of 1 mM HCl for 1 hour, centrifuged at 1000 × g for 5 minutes at RT, and washed for 15 minutes with 10 ml of 1 mM HCl followed by centrifugation. Swelled sepharose was washed with 2 × 10 ml of coupling buffer (100 mM NaHCO_3_, 500 mM NaCl, pH 8.3) and centrifuged between each wash. Then, 10 mg of StcE E447D was diluted with coupling buffer to a final volume of 10 ml, added to the swelled CNBr-activated sepharose, and rotated overnight at 4°C. The sepharose was washed with 2 × 10 ml of coupling buffer and quenched with 10 ml ice-cold 250 mM glycine on overnight rotation at 4°C. The sepharose was washed five times with 10 ml of coupling buffer and resuspended in 10 ml of H buffer (150 mM NaCl, 50 mM Tris pH 7.4 + 0.02% NaN_3_). The volume of the sepharose was adjusted to a 50% slurry. And 50–100 μl of 50% StcE-CnBr slurry was used for each pull-down.

### EndoH, PNGaseF, and StcE treatments

3 × 10^5^ Caco-2 cells were seeded in 9.6-mm^2^ dishes and differentiated for 14 dpc. Cells washed 3 × 5 minutes with PBS at RT and lysed with 200 μl of ice-cold lysis buffer (25 mM Tris-HCL pH 7.4, 150 mM NaCl, 4% glycerol, 1% Triton X-100) complemented with a final concentration of 1X EDTA-free complete protease inhibitor cocktail (34044100, Roche) and 1 mM PMSF (78830, Sigma-Aldrich). Cells were incubated with lysis buffer for 10 minutes on ice, collected by scraping, and homogenized by sonication in an ice-cold water bath at 40% amplitude for 30-second pulses for 4 minutes. Cell lysates were cleared by centrifugation at 16,000 × g for 30 minutes at 4°C. For EndoH and PNGaseF treatments, 30 μl of cell lysates were mixed with 10 μl of 200 mM DTT, 1 μl of PMSF, 2 μl of 3.0M NaAc pH 5.4 (only for EndoH treatment), 5 μl of EndoH (11643053001, Sigma) or 6 μl of PNGaseF (11365177001, Sigma) and diluted with lysis buffer to 50 μl. Untreated control samples were prepared without the addition of NaAc, EndoH, or PNGaseF. For StcE treatment, 42 μl of cell lysate was mixed with 1 μl of active StcE (5.8 mg/ml). All samples were incubated at 37°C overnight and reduced in 4X reducing sample buffer (8% SDS, 400 mM dithiothreitol, 40% glycerol, 200 mM Tris pH 6.8, 0.4% bromophenol blue) followed by boiling at 95°C for 5 minutes.

### Immunoblots and in-gel western blots

Samples were separated on precast 4–12% SDS polyacrylamide gel (XP04125BOX, Thermo Fisher Scientific). Proteins were transferred to a PVDF-FL membrane (05317, Millipore) with a current of 2.5 mA/cm^2^ for 1 hour. The membrane was blocked in 5% non-fat milk in PBS for 30 minutes and incubated with primary antibodies diluted in 5% non-fat milk in PBS + 0.1% Tween-20 (PBS-T) overnight at 4°C. The membrane was washed three times in PBS-T and incubated with secondary antibodies diluted in 5% non-fat milk in PBS-T + 0.02% SDS for 1 hour at RT in the dark. The membrane was washed three times in PBS-T and visualized on an Odyssey CLx system (LI-COR Biosciences). Protein quantification was performed using Image Studio quantification software (LI-COR Biosciences). For Coomassie stains, membranes were stained with Imperial Protein Stain (24615, Thermo Scientific), destained in 5% MeOH and 7% acetic acid, and visualized on an Odyssey CLx near-infrared fluorescence imaging system (LI-COR Biosciences). Samples for in-gel westerns were separated on precast 4–12% SDS polyacrylamide gels. Proteins were fixed in the gel using 50% isopropanol + 5% acetic acid in ultrapure water for 15 minutes. The gel was washed extensively in ultrapure water for 3 × 15 minutes and incubated with primary antibodies diluted in 5% BSA in PBS overnight at 4°C. After 3 × 10-minute washes in PBS-T, the gel was incubated with secondary antibodies diluted in 5% BSA in PBS + 0.1% Tween^®^ 20 for 2 hours (RT). The gel was washed 3 × 10 minutes in PBS-T and visualized on an Odyssey CLx system. Raw non-cropped immunoblots are provided in Supplementary Table S3.

### Biotin proximity labeling by antibody recognition

3 × 10^5^ Caco-2 cells seeded on 9.6-mm^2^ wells and differentiated for 14 days were washed two times with PBS. Cells were fixed with 4% paraformaldehyde (PFA) in PBS for 15 minutes at RT and washed twice in PBS-T. Next, cells were permeabilized in PBS + 0.5% Triton X-100 for 7 minutes at RT followed by 3 × 10-minute washes with PBS-T. And 30 mM H_2_O_2_ in PBS was added overnight at RT to quench endogenous peroxidase activity. Another 30 mM of fresh H_2_O_2_ in PBS was added for 10 minutes followed by two 10-minute washes with PBS-T. Cells were incubated with blocking buffer (5% BSA in PBS) for 2 hours on a shaker and stained with Flag mAb 1:500 diluted in blocking buffer overnight at 4°C in a humid chamber on an orbital shaker. After three subsequent 1 hour PBS-T washes, cells were incubated with 1:1000 goat anti-mouse HRP diluted in blocking buffer for 1 hour at RT. Unbound antibody was removed by three 2-hour washes with PBS-T and the cells were pre-incubated with 500 μM biotin-tyramide (final concentration) at RT. After 10 minutes, a final concentration of 2.5 mM H_2_O_2_ in PBS was added to the biotin-tyramide solution for 2 minutes at RT. To quench the reaction, 3 × 5-minute washes in 500 μl of 500 mM sodium ascorbate was used followed by 3 × 10-minute washes with PBS-T. Cells were lysed in 200 μl PBS-T + 2% SDS + 2% deoxycholate + 1X complete protease inhibitor. The cells were collected by scraping, sonicated for 10 seconds, and boiled at 95°C for 60 minutes. Samples were cleared by centrifugation at maximum speed for 10 minutes. The supernatants were diluted in 1 ml PBS-T, and 50 μl was saved as an input control. Next, 20 μl of pre-washed Streptavidin Dynabeads (11,205D, Thermo Fisher Scientific) was added to each sample and incubated for 48 hours, rotating at 4°C. Beads were washed in (1) 15 ml PBS-T, (2) 15 ml PBS-T + 1 M NaCl, (3) 15 ml PBS, and (4) 15 ml of PBS + 0.5% Triton X-100. Beads were re-suspended in PBS, and proteins were eluted by adding 1 vol of 2X lysis buffer (4% SDS, 200 mM DTT, 125 mM Tris HCl pH 6.8) and boiling at 95°C for 5 minutes. The eluate was separated from beads using Costar Spin-X filter units.

### Sample preparation for LC-MS/MS

Eluted proteins from light and heavy samples prepared with method 1 were mixed at a 1:1 ratio and added onto a 10 kDa cutoff filter (OD010C33, PALL) followed by the addition of 1 μl of 1 mM DTT. Eluates prepared by method 2 were directly added to the cut-off filters. Proteins were digested with trypsin overnight at 37°C using filter-aided sample preparation (FASP) [48]. Peptide concentration after elution was measured at 280 nm using NanoDrop (Thermo Fisher Scientific), and peptides were cleaned with StageTip C18 columns [49] before mass-spectrometry (MS) analysis.

Eluates from proximity labeling experiments were reduced at 37°C for 60 minutes with DTT at 100 mM final concentration and further processed using the modified FASP method. In short, the samples were diluted 1:4 v/v by 8M urea solution, transferred onto Microcon-30kDa centrifugal units (Merck Millipore, Carrigtwohill, Ireland), and washed with 8 M urea and with digestion buffer (0.5% sodium deoxycholate (SDC) in 50 mM TEAB. Free cysteine residues were modified using 10 mM methyl methanethiosulfonate solution in digestion buffer for 20 minutes at room temperature, and the filters were washed twice with 100 μl of digestion buffer. Proteins were digested overnight at 37°C by adding 0.3 μg of Pierce trypsin protease (MS grade, Thermo Fisher Scientific), followed by a second incubation with 0.3 μg trypsin for three hours.

Peptides were collected by centrifugation and labeled using Tandem Mass Tag 10plex reagent (90,061, Thermo Fischer Scientific) according to the manufacturer’s instructions. The labeled samples were combined into one pool, concentrated using vacuum centrifugation, and SDC was removed by acidification with 10% TFA and subsequent centrifugation. The digested peptides were cleaned using the HiPPR detergent removal resin kit (PN 88305, Thermo Fisher Scientific, Waltham, MA, USA) according to the manufacturer’s instructions. The sample was subsequently separated into five fractions on Pierce High pH Reversed-Phase spin column kit (Thermo Fisher Scientific) using stepwise elution with 0.1% aqueous trimethylamine solution containing 10% to 50.0% of acetonitrile. The fractions were dried and reconstituted in 15 μl of 3% acetonitrile + 0.2% formic acid for LC-MS/MS analysis.

### Liquid chromatography-MS/MS

Nano-LC-MS/MS for SILAC samples was performed on a Q-Exactive HF mass-spectrometer (Thermo Fischer Scientific), connected with an EASY-nLC 1000 system (Thermo Fischer Scientific) through a nanoelectrospray ion source. Peptides were loaded on a reverse-phase column (150 mm^3^ 0.075-mm inner diameter, New Objective, New Objective, Woburn, MA) packed in-house with Reprosil-Pur C18-AQ 3-mm particles (Dr. Maisch, Ammerbuch, Germany). Peptides were separated with a 50-minute gradient: from 5 to 30% B in 35 minutes, 30 to 45% B in 5 minutes, and 45 to 100% B in 1 minute, followed by 9-minute wash with 100% of B (A, 0.1% formic acid; B, 0.1% formic acid/80% acetonitrile) using a flow rate of 250 nl/min. Q-Exactive HF was operated at 250°C capillary temperature and 2.0 kV spray voltage. Full mass spectra were acquired in the Orbitrap mass analyzer over a mass range from m/z 350 to 1600 with a resolution of 60,000 (m/z 200) after the accumulation of ions to a 3 × e^6^ target value based on predictive AGC from the previous full scan. Fifteen most intense peaks with a charge state ≥ 2 were fragmented in the higher-energy collision dissociation (HCD) cell with a normalized collision energy of 27%, and the tandem mass spectrum was acquired in the Orbitrap mass analyzer with a resolution of 15,000 after accumulation of ions to a 1 × e^5^ target value. Dynamic exclusion was set to 20 seconds. The maximum allowed ion accumulation times were 20 ms for full MS scans and 50 ms for tandem mass spectrum.

The tandem mass tag (TMT)-labeled fractions were analyzed on an Orbitrap Fusion Lumos Tribrid mass spectrometer interfaced with an Easy-nLC 1200 liquid chromatography system (both Thermo Fisher Scientific). Peptides were trapped on an Acclaim Pepmap 100 C18 trap column (100 μm × 2 cm, particle size 5 μm, Thermo Fischer Scientific) and separated on an analytical column (75 μm × 35 cm, packed in-house with Reprosil-Pur C18, particle size 3 μm, Dr. Maisch, Ammerbuch, Germany) using a linear gradient from 5% to 33% B over 77 minutes followed by an increase to 100% B for 3 minutes and 100% B for 10 minutes at a flow of 300 nl/min. Solvent A was 0.2% formic acid in water and solvent B was 80% acetonitrile, 0.2% formic acid. MS scans were performed at 120,000 resolution in the m/z range 375–1375. The most abundant doubly or multiply charged precursors from the MS1 scans were isolated using the quadrupole with 0.7 m/z isolation window with a ‘top speed’ duty cycle of 3 seconds and dynamic exclusion within 10 ppm for 45 seconds. The isolated precursors were fragmented by collision-induced dissociation at 35% collision energy with a maximum injection time of 50 ms and detected in the ion trap, followed by multinotch (simultaneous) isolation of the top 10 MS2 fragment ions within the m/z range 400–1400, fragmentation (MS3) by HCD at 65% collision energy, and detection in the Orbitrap at 50,000 resolution, m/z range 100–500, and maximum injection time 105 ms.

### MS data analysis

MS raw files from SILAC experiments were processed with MaxQuant software version 1.5.7.4 [50]; peak lists were identified by searching against the human UniProt protein database (downloaded 2019.04.16) supplemented with an in-house database containing all the human mucin sequences (http://www.medkem.gu.se/mucinbiology/databases/). Searches were performed using trypsin as an enzyme, with a maximum of two missed cleavages, a precursor tolerance of 20 ppm in the first search used for recalibration, followed by 7 ppm for the main search and 0.5 Da for fragment ions. Carbamidomethylation of cysteine was set as a fixed modification. Methionine oxidation, protein N-terminal acetylation, and 3-(carbamidomethyl-thio)propanoyl (DSP cross-linker) were set as variable modifications. Arg6 and Lys6 were chosen as label modifications. The required false discovery rate (FDR) was set to 1% both for peptide and protein levels and the minimum required peptide length was set to seven amino acids.

SILAC data were analyzed with Perseus (version 1.5.5.0). First, proteins identified in the decoy database were removed together with proteins only identified by site and common contaminants. Heavy and light intensities were log_2_ transformed and filtered based on valid values in at least one group (heavy or light). Missing values were imputed based on the normal distribution of measured values using default values (width = 0.3 and downshift = 1.8). Significantly enriched proteins were determined with a two-sided t-test and permutation-FDR = 0.05, S0 = 0.1, and 250 randomizations. These proteins were also manually validated as previously described [51].

Identification and relative quantification of TMT samples from proximity labeling was performed using Proteome Discoverer version 2.4 (Thermo Fisher Scientific). The database search was performed using the Mascot search engine v. 2.5.1 (Matrix Science, London, UK) against the Swiss-Prot Homo sapiens database. Trypsin was used as a cleavage rule with no missed cleavages allowed; methyl thiolation on cysteine residues and TMT at peptide N-termini and on lysine side chains were set as static modifications, and oxidation on methionine was set as a dynamic modification. Precursor mass tolerance was set at 5 ppm and fragment ion tolerance at 0.6 Da. Percolator was used for the peptide-spectrum match (PSM) validation with a strict FDR threshold of 1%. Quantification was performed in Proteome Discoverer 2.4. The TMT reporter ions were identified with 3 mmu mass tolerance in the MS3 HCD spectra and the TMT reporter S/N values for each sample were normalized within Proteome Discoverer 2.4 on the total peptide amount. Only the unique identified peptides were considered for the protein quantification.

### Immunofluorescence

Staining of Caco-2 cells grown on chamber slides (154534PK, Thermo Fisher Scientific) was performed as described previously [24]. In brief, cells were washed three times in warm PBS followed by 10-minute fixation in 4% PFA in PBS at RT. Excess PFA was washed away with three PBS washes and cells permeabilized by 0.1% Triton X-100 in PBS for 7 minutes. After permeabilization, cells were washed three times with PBS and blocked overnight in 5% BSA in PBS at 4°C. Cells were incubated with primary antibodies diluted in 5% BSA in PBS for 2 hours at 24°C and then washed three times with PBS and incubated with secondary antibodies for 1 hour at RT. After three washes with PBS, cell nuclei were stained with Hoechst for 7 minutes at RT. Chamber slides were washed three times with PBS and mounted with Prolong anti-fade (Invitrogen).

Harvested ileum was fixed in Carnoy’s fixative (60% absolute methanol, 30% chloroform, and 10% glacial acetic acid) or 4% PFA solution. Samples fixed in Carnoy’s fixative were embedded in paraffin. Paraffin-embedded sections were deparaffinized in xylene substitute (2, 3, and 10 minutes, 60°C) and rehydrated in 100% ethanol (10 minutes), 70% (v/v) ethanol (5 minutes), 50% (v/v) ethanol (5 minutes), and 30% (v/v) ethanol (5 minutes). Sections were placed in antigen retrieval buffer (0.01 M citric acid, pH 6.0) at 100 degrees for 10 minutes and then brought to RT (2 hours) and transferred to PBS. Tissues were enclosed with a PAP pen followed and blocked with 5% fetal calve serum (FCS) in PBS for 20 minutes at RT. Primary antibodies were diluted in 5% FCS in PBS and incubated overnight at 4°C. After 3 × 5-minute washes in PBS, sections were incubated with secondary antibodies diluted in 5% FCS in PBS. DNA was stained with Hoechst for 5 minutes at RT. Coverslips were mounted using Prolong Gold antifade (P36980, Thermo Fisher Scientific) and polymerized overnight at RT in the dark. Images and Z-stacks were acquired on a Zeiss LSM 700 (Plan-Apochromat 40 ×/1.3 Oil DIC M27 lens and 1.58μs pixel dwell) and a Zeiss LSM900 (equipped with an Airyscan2 detector and plan-Apochromat 63 ×/1.4 Oil DIC M27 lens).

### Image analysis

All image analysis and processing were performed in ImageJ software v.1.53.t (National Institutes of Health, Bethesda, MD) if not otherwise stated. Confocal images are shown as maximum projections except from the YZ and XZ orthogonal views when stated in the manuscript. Cellular height was quantified from YZ-projections of confocal images. Line intensity profiles were generated from z-axis profiles for each individual channel, averaged to 15-μm distance from the basolateral to the apical membrane, and normalized to values between 0 and 100. Basolateral localization of endogenous MUC17 was quantified using the Measure analysis tool by capturing the integrated intensity (IntDen) of fluorescence overlapping with basolateral membrane. Quantification of basolateral localization of 3F-MUC17(7TR) in Caco-2 WT and KO cells was performed by calculating the area under the curve (AUC) for the non-normalized line intensity profiles. The basolateral compartment of each cell was defined as the AUC below 50% of the peak maxima of the DNA reference stain. The MUC17 AUC below this level corresponding to basolateral MUC17 was normalized to the total MUC17 AUC and is expressed as percentage. Intensity profiles were used to calculate the distance (μm) of the maximum mean fluorescent intensity for every protein. The distance for proteins spatially separated in a cell will differ greatly, while proteins in proximity will result in a similar distance. Fractional overlap between endogenous Muc17 and Myo1b or Snx27 in ileal sections was calculated using segmentation followed by subtraction using the Image calculator function in ImageJ. Co-localization coefficients throughout Z-stacks were obtained using ZEN 2010 software. The fractional overlap of high-magnification images was quantified by Isosurface segmentation and the shortest distance function in the Imaris software v.9.5 (Oxford Instruments).

### Transmission electron microscopy

3F-MUC17(7TR) Caco-2 cells grown on Transwell filters (CLS3496, Merck) for 7, 14, and 21 dpc were fixed in primary fixative (1.33% glutaraldehyde in water, 0.1 M cacodylate buffer, 0.05% Ruthenium Red in water) for 1 hour at RT. Fixed cells were pre-washed in 0.05 M cacodylate buffer and then washed extensively in 2 × 10-minute 0.05 M cacodylate buffer, 20-minute 0.05 M cacodylate buffer + 0.02 M glycine followed by 2 × 10-minute 0.05 M cacodylate buffer. Secondary fixative (1.33% osmium tetroxide in water, 0.1 M cacodylate buffer, 0.05% Ruthenium Red in water) was added to the cells for 1 hour and incubated at 4°C in the dark on an orbital shaker. The secondary fixative was removed by a few quick washes in water followed by 6 × 5-minute washes in water. Cells were incubated with tertiary fixative (1% filtered aqueous uranyl acetate) for 30 minutes at room temperature in the dark followed by a few washes with water. Excised membranes were stained in lead aspartate (0.02 M lead nitrate and 0.03M aspartic acid pH 5.5) for 20 minutes at RT. Cells were washed three times in water, incubated overnight in water followed by another three washes in water. Dehydration of cells was done in a series of ethanol solutions at RT (5-minute 30% EtOH, 5-minute 50% EtOH, 5-minute 70% EtOH, 5-minute 85% EtOH, 5-minute 95% EtOH, 5 × 5-minute 100% EtOH). The cells were embedded in Hard-Plus Epoxy 812-resin (14115, Electron Microscopy Sciences, U.S.A.) at RT as follows: 25% resin in acetone for 1 hour, 50% resin for 2 hours, 75% resin for 1 hour, 100% resin for 3 × 30 minutes, and 100% resin overnight on an orbital shaker. After another incubation of 2 × 1 hour 100% resin, samples were incubated with 100% resin + accelerator (240 μl/10 ml) for 1 hour and another 100% + accelerator for several hours. Infiltrated samples were embedded in resin-silicon free molds with resin + accelerator and let to polymerize for 48 hours at 60°C. Transversal ultrathin sectioning (70–90 nm) of cells from the apical to the basolateral membrane was performed on Leica UC6 Ultracut, collected on copper 100 hexagonal mesh support grids, and post-stained with Reynold’s solution at RT for 5 minutes in a sealed chamber with NaOH-pellets. Sections were imaged on TEM FEI Talos (Thermo Fisher, U.S.A) equipped with a 4k ×4k Ceta CMOS camera operating at 120 kV with LaB6 filament.

### Fluorescence recovery after photobleaching

StcE E447D was labeled with CF 555 Succinimidyl Ester (92214, Biotium) according to the manufacturer’s protocol. 500 μl of serum-free medium containing 10 μg/ml of CF 555-StcE E447D was added to WT and knockout Caco-2 cells differentiated on 30-mm plates for 30 minutes at RT. After two washes with 3 ml PBS at RT, images were acquired using LSM700 with a plan-apochromat × 20/1.0 DIC M27 75-mm water objective (Zeiss) at 1.5 digital zoom and ZEN 2010 software. Photobleaching at 100% laser power for a duration of 25 μs was performed after five initial scans (pixel dwell per scan) using the 555 nm laser. Four 5 μm × 5 μm square regions of interest (ROI) were selected according to the following scheme: ROI1, analyze in cell 1 (bleach control), and ROI2, bleach and analyze in cell 2. Recovery images were acquired every 2 seconds for 12 minutes. Raw data are analyzed by the formula:

(1) $$\text{Norm}(t)=\frac{Ref_{\text{pre}\;-\;\text{bleach}}}{ref(t)}\cdot \frac{FRAP_{\left(t\right)}}{FRAP_{\text{pre}\;-\;\text{bleach}}}$$

(2) $$Norm_{0-1}(t)=Norm_{\text{min}}-\text{Norm}(t)$$

Where *Ref*_*pre-bleach*_ is the mean intensity of ROI1 before bleaching, *FRAP*_*pre-bleach*_ is the mean intensity of the ROI2 pre-bleaching, *ref(t*) is the intensity of ROI1 at time point *t*, and *FRAP(t*) is the intensity of ROI2 at time point *t. Norm(t*) represents fluorescence in ROI2 at time-point *t,* which was corrected for bleaching during analysis recovery. *Norm*_*0–1*_*(t*) sets the mean intensity of ROI2 before bleaching to 1 and after bleaching to 0. Recovery halftimes and mobile fractions were extracted by fitting the data to a non-linear curve based on a one-phase association.

### Quantification and statistical analysis

Data analysis was performed using GraphPad Prism (version 9.5) and Perseus (version 1.5.5.0). Graphs were prepared using either Perseus (version 1.5.5.0) or GraphPad Prism (version 9.5). Venn Diagrams were created using: https://bioinformatics.psb.ugent.be/webtools/Venn/. One- or two-way ANOVA followed by Tukey’s or Sidak’s multiple comparisons test or Kruskal Wallis and Dunn’s multiple comparisons test was done for comparisons of multiple groups. The unpaired t-test with Welch’s correction, assuming non-equal SDs, was used for the comparison of two groups: *P < 0.05, **P < 0.01, ***P < 0.001, and ****P < 0.0001.

## Supplementary Material

Supplementary Tables

Supplementary Figures

## Figures and Tables

**Table 1:** Antibodies and fluorescent probes.

Antibody	Source	Method	Working dilution
Anti-Actin	MAB1501, Sigma-Aldrich	IB	1:5000
Anti-CDHR5	HPA009081, Sigma-Aldrich	ICC/IHC	1:250
Anti-Ezrin	E8897, Sigma-Aldrich	ICC/IHC	1:500
Anti-Ezrin	HPA021616, Atlas Antibodies	ICC/IHC	1:100
Anti-Flag	F1804, Sigma-Aldrich	ICC/IHC	1:500
		IB	1:2000
		In-gel	1:500
Anti-GFP	G6539, Sigma-Aldrich	IB	1:1000
Anti-HA	H3663, Sigma-Aldrich	ICC/IHC	1:500
		IB	1:2000
Anti-MYOIB	HPA060144, Atlas antibodies	ICC/IHC	1:32
		IB	1:1000
Anti-SNX27	ab77799, Abcam	ICC/IHC	1:100
		IB	1:1000
Anti-MUC17-C1	In-house antibody	IB	1:3000
Anti-MUC17-S1	In-house antibody	IB	1:250
Anti-MYO5B	HPA040902, Atlas antibodies	IB	1:350
Alexa Fluor-488 donkey anti-mouse	A2102, Thermo Fisher Scientific	ICC/IHC	1:300
Alexa Fluor-555 donkey anti-mouse	A31572, Thermo Fisher Scientific	ICC/IHC	1:300
Alexa Fluor-488 goat anti-rabbit	A11055, Thermo Fisher Scientific	ICC/IHC	1:300
Alexa Fluor-555 goat anti-rabbit	A21434, Thermo Fisher Scientific	ICC/IHC	1:300
Alexa Fluor-647 Phalloidin	A22267, Thermo Fisher Scientific	ICC/IHC	1:150
Donkey anti-mouse Alexa Fluor 680	A10038, Thermo Fisher Scientific	IB	1:20,000
		In-gel	1:10,000
Goat anti-rabbit Alexa Fluor 790	A11369, Thermo Fisher Scientific	IB	1:20,000
		In-gel	1:10,000
Hoechst 34,580	H21486, Thermo Fisher Scientific	ICC/IHC	1:10,000

IB, immunoblotting. ICC, Immunocytochemistry. IHC, Immunohistochemistry.

## Data Availability

The proteomics data have been deposited to the ProteomeXchange Consortium (http://proteomecentral.proteomexchange.org) via the PRIDE partner repository with the dataset identifier PXD058694.

## Abbreviations

ANOVA: analysis of variance
AP: affinity purification
AUC: area under the curve
CNBr: cyanogen bromide
DSP: dithiobis(succinimidyl propionate)
DTT: DL-dithiothreitol
FASP: filter-aided sample preparation
FCS: fetal calve serum
FDR: false discovery rate
FRAP: fluorescence recovery after photobleaching
HCD: higher-energy collision dissociation
IB: immunoblotting
IMAC: intermicrovillar adhesion complex
IMDM: Iscove’s modified Dulbecco’s medium
MMTS: methyl methanethiosulfonate
MS: mass-spectrometry
MVID: Microvillus inclusion disease
PBM: PDZ-binding motif
PBS-T: PBS + 0.1%Tween-20
PDZ: PSD95, DLG1, ZO-1
PFA: paraformaldehyde
PFA: paraformaldehyde
PSM: peptide-spectrum match
ROI: regions of interest
Re-CLIP: reversible cross-link immunoprecipitation
SDC: sodium deoxycholate
SEA: sperm protein, enterokinase, and agrin
SILAC: stable isotope labeling in cell culture
TEM: transmission electron microscopy
TRs: tandem repeats
cDNA: Copy DNA
dpc: days post confluency
