# Supplementary material for: The MYO1B and MYO5B motor proteins and the sorting nexin SNX27 regulate apical targeting of membrane mucin MUC17 in enterocytes
Source: Biochem J. 2025 Jan 8;482(1):BCJ20240204. doi: 10.1042/BCJ20240204 (PMC12172626; doi:10.1042/BCJ20240204)
Supplement: online supplementary figure 1. [file bcj-482-1-BCJ20240204-s001.docx]

**Supplementary material**

**The MYO1B and MYO5B motor proteins and the SNX27 sorting nexin regulate membrane mucin MUC17 trafficking in enterocytes**

Sofia Jäverfelt^1^, Gustaf Hellsén^1^, Izumi Kaji^2^, James. R. Goldenring^2^, Thaher Pelaseyed^1^*

1. Department of Medical Biochemistry and Cell Biology, Institute of Biomedicine, University of Gothenburg, Box 440, 405 30 Gothenburg, Sweden.

2. Department of Cell and Developmental Biology, Vanderbilt University, Nashville, TN 37232, USA; Epithelial Biology Center, Vanderbilt University Medical Center; Section of Surgical Sciences, Vanderbilt University Medical Center, Nashville, TN 37232, USA; Nashville VA Medical Center, Nashville, TN 37232, USA.

* Corresponding author: [thaher.pelaseyed@medkem.gu.se](mailto:thaher.pelaseyed@medkem.gu.se)

*Running title: Trafficking machinery of MUC17*

**
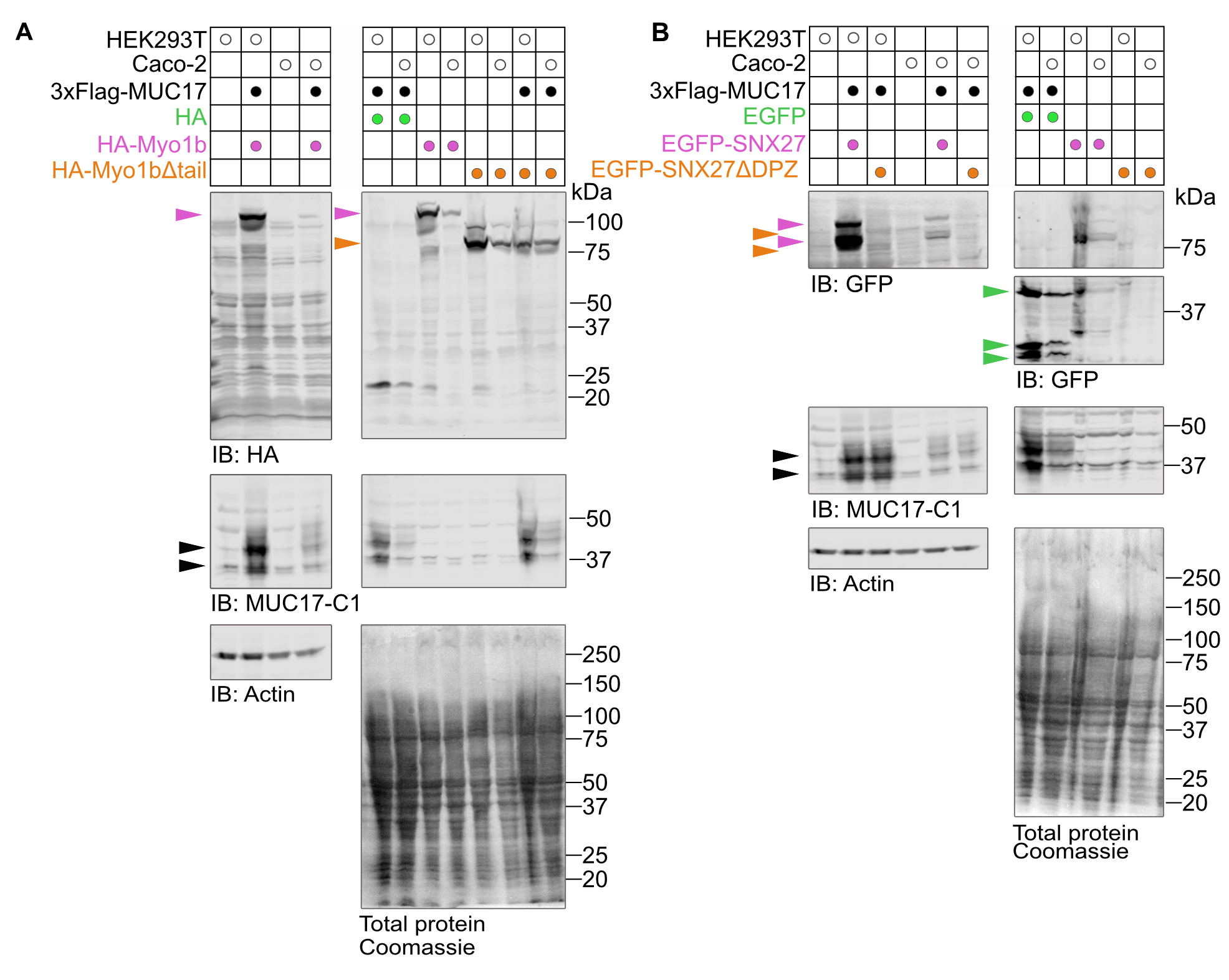
Figure S1. Expression of recombinant 3F-MUC17(7TR), HA-Myo1b, EGFP-SNX27 in HEK293 and Caco-2 cells.**

A) Immunoblots of HEK293T and Caco-2 cells expressing 3F-MUC17(7TR), HA, HA-Myo1b, and HA-Myo1bΔtail. The arrowheads in magenta point to HA-Myo1b, orange arrowheads point to HA-Myo1b∆tail, and black arrowheads point to the C-terminal fragment of MUC17.

B) Immunoblots of HEK293T and Caco-2 cells expressing combinations of 3F-MUC17(7TR), EGFP, EGFP-SNX27, and EGFP-SNX27ΔPDZ. The arrowheads in magenta point to isoforms of EGFP-SNX27, orange arrowheads point to isoforms of EGFP-SNX27∆PDZ, and black arrowheads point to the C-terminal fragment of MUC17.

**
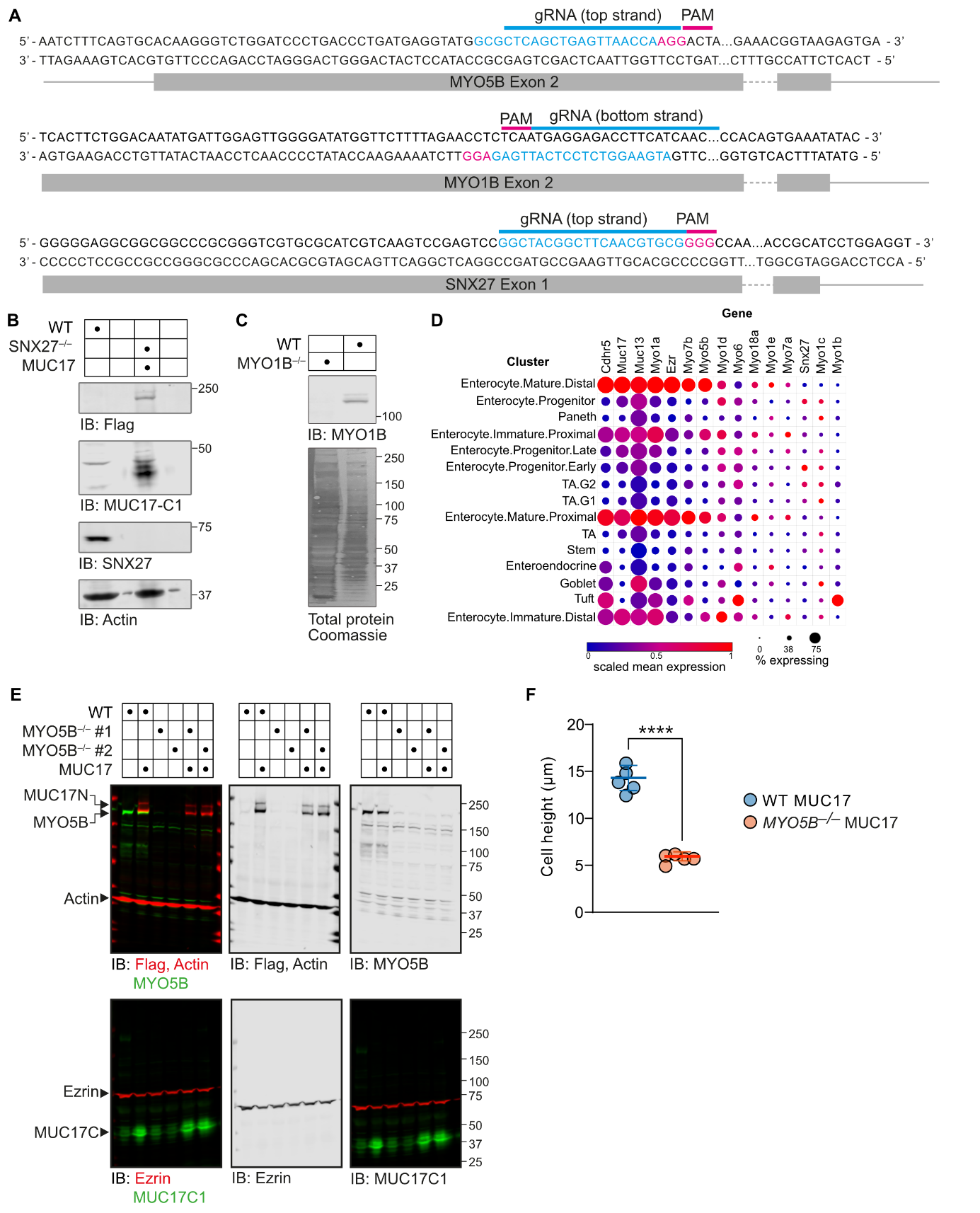
Figure S2. CRISPR/Cas9-mediated deletion of *MYO5B*, *MYO1B*, and *SNX27* genes in Caco-2 cells.**

A) Placement of CRISPR/Cas9 guide RNAs for the deletion of human *MYO5B*, *MYO1B*, and *SNX27* genes in Caco-2 cells.

B) Validation of CRISPR/Cas9-mediated deletion of *SNX27* in Caco-2 cells. Actin is used as a loading control.

C) Validation of CRISPR/Cas9-mediated deletion of *MYO1B* in Caco-2 cells. Coomassie staining shows total protein amounts in lysate from each cell line.

D) Single-cell transcriptomics from murine small intestine [1] showing non-muscle myosins and core microvillar components identified in [2]. Graphical visualization was prepared using the Broad Institute Single Cell Portal.

E) Validation of CRISPR/Cas9-mediated deletion of *MYO5B* in WT and 3F-MUC17(7TR)- expressing Caco-2 cells. Ezrin is used as a loading control.

F) Cellular height (µm) of WT 3F-MUC17(7TR) and *MYO5B^–/–^* 3F-MUC17(7TR) Caco-2 cells, n = 5 scans. Data are presented as mean ± SD. ****p<0.0001 as determined by one-way ANOVA with Dunnett´s multiple comparisons test.

**Figure S3. Expression of 3F-MUC17(7TR) in WT and mutant Caco-2 cells.**

Immunoblot of total 3F-MUC17(7TR) and total protein stained with Coomassie for WT and *MYO1B^−/−^*, *MYO5B^−/−^* and *SNX27^−/−^* Caco-2 cells used for quantification in figure 7A.


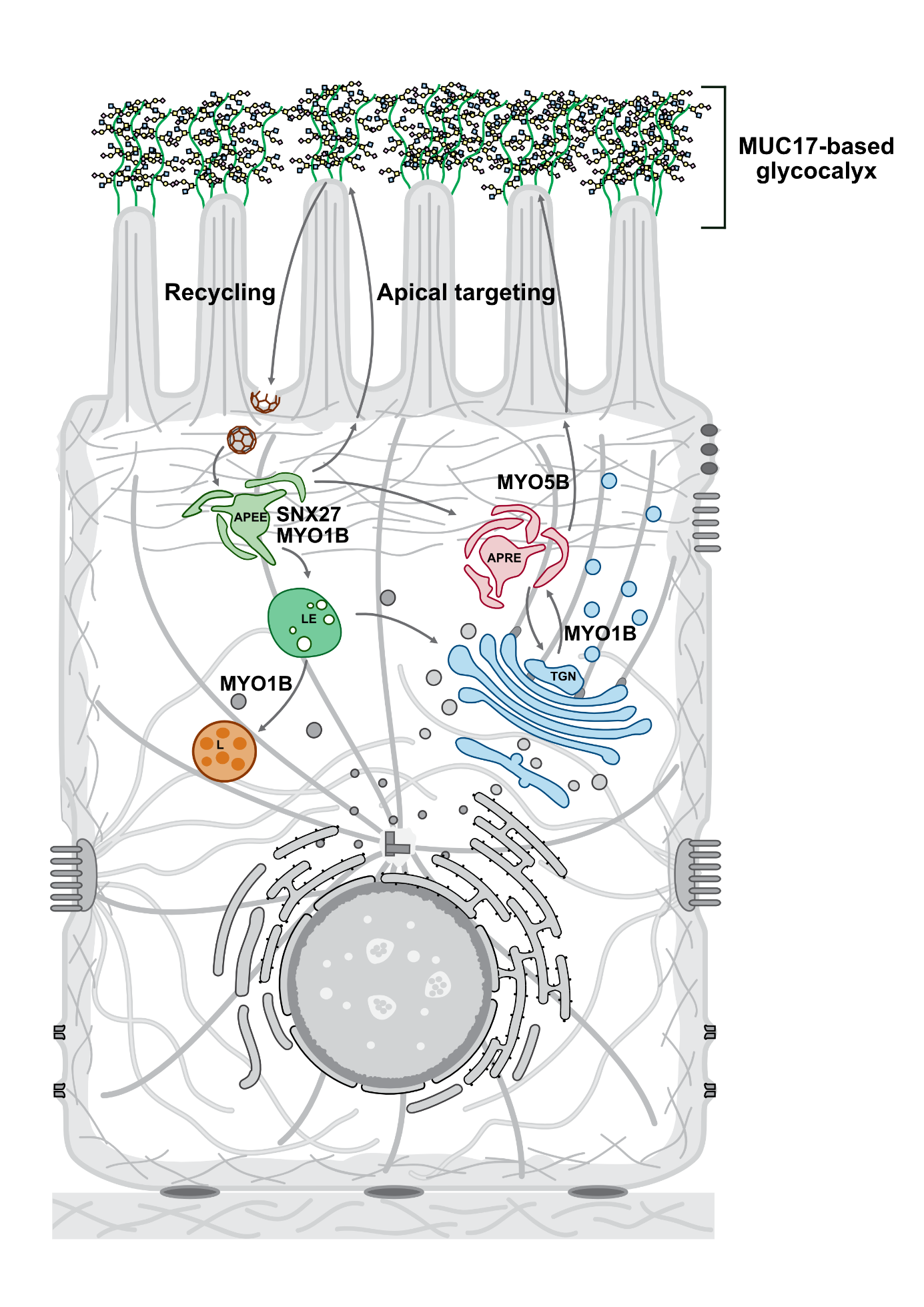


**Figure S4. Proposed model for MUC17 apical targeting in polarized epithelial cells.**

APEE; apical early endosome, LE: late endosome, L; lysosome, APRE; apical recycling endosome, TGN; trans-Golgi network

**Figure S5. cDNA and primers used for cloning of recombinant MUC17.**

A) The cDNA sequence used for generating recombinant 3F-MUC17(7TR).

B) Forward and reverse primer combinations for generation of 3F-MUC17(7TR) with overhangs for subsequent cloning into the pXL-CAG-Zeocin-3xF2A plasmid.

**References:**

1. Haber, A.L., et al., *A single-cell survey of the small intestinal epithelium.* Nature, 2017. **551**(7680): p. 333-339.

2. Layunta, E., et al., *IL-22 promotes the formation of a MUC17 glycocalyx barrier in the postnatal small intestine during weaning.* Cell Rep, 2021. **34**(7): p. 108757.
